# Rheumatic heart disease burden, trends, and inequalities in Asia, 1990–2019

**DOI:** 10.1080/16549716.2023.2215011

**Published:** 2023-05-26

**Authors:** Chengfu Guan, Wenlin Xu, Shuyi Wu, Jinhua Zhang

**Affiliations:** Department of Pharmacy, Fujian Maternity and Child Health Hospital, College of Clinical Medicine for Obstetrics & Gynecology and Pediatrics, Fujian Medical University, Fuzhou, China

**Keywords:** Cardiology, global burden of diseases, global health, rheumatic heart disease, health inequalities

## Abstract

**Background:**

Rheumatic heart disease (RHD) is a serious global public health problem.

**Objectives:**

This study aims to characterise the regional burden, trends, and inequalities of RHD in countries and territories in the Asian Region.

**Methods:**

The RHD disease burden was measured in terms of the numbers of cases and deaths, prevalence, disability-adjusted life years (DALYs), disability-loss healthy life years (YLDs), and years of life lost (YLLs) in 48 countries in the Asian Region. Data on RHD were extracted from the 2019 Global Burden of Disease. This study analysed changing trends in the burden between 1990 and 2019, quantified regional inequalities in mortality, and classified countries by 2019 YLLs.

**Results:**

There were an estimated 22 246 127 cases of RHD in the Asian Region in 2019 and 249 830 deaths. The prevalence of RHD in the Asian Region in 2019 was 9% lower than the global estimate, while mortality was 41% higher. The mortality rate for RHD in the Asian Region trended downwards from 1990 to 2019, with an average annual percentage change of −3.2% (95% UI −3.3 to −3.1). From 1990 to 2019, absolute inequality in RHD-related mortality decreased in the Asian Region while relative inequality increased. Of the 48 countries studied, twelve had the highest level of RHD YLLs in 2017 and the smallest reduction in YLLs from 1990 to 2019.

**Conclusion:**

Although the burden of RHD in the Asian Region has steadily decreased since 1990, it remains a serious public health issue requiring greater attention. In the Asian Region, inequalities in the distribution of the RHD burden remain significant, with economically deprived countries typically bearing a greater share of the load.

## Introduction

Rheumatic heart disease (RHD) is a chronic valve disease that results from valve damage caused by an abnormal immune response to group A streptococcal infection [1,2]. RHD is a global public health issue, with earlier research indicating that there were roughly 33.4 million instances of RHD and 319,400 fatalities from RHD worldwide in 2015 [3]. The burden of RHD is related to factors such as residential environment and socioeconomic status and mainly affects low- and middle-income countries, especially vulnerable groups in these countries [4,5]. The disease is a particularly common cardiovascular disease in children and adolescents and is a major cause of cardiovascular death and disability [3,6,7]. In addition, repeated hospitalisations and heart valve repair or replacement for RHD patients due to valve damage have social and economic impacts on families and societies more broadly [8]. The World Health Organization’s global health estimates released in 2020 indicate that the contribution of RHD to overall global mortality has barely declined between 2000 and 2019 [9]. Despite RHD’s enormous global public health burden, there have been few publications and conference reports on the disease, and media coverage has been limited [10,11]. A review of the literature on the prevalence of RHD in the Asian Region shows that studies on the prevalence of RHD have focused on South-Central Asian countries such as India, Bangladesh, and Pakistan. Evidence on RHD in other Asian countries is deficient [12]. We, therefore, conducted this study to comprehensively assess the trends and burdens of RHD in 48 countries in the Asian Region (see Appendix Table A7).

Equity has long been recognised as an important goal of the health sector. However, health inequalities still exist between countries and between different social classes within countries [13]. Health inequality-related research and interest have increased in recent years [13–15]. RHD is known as the ‘disease of the poor’, and the burden is strongly related to economic issues. The Asian Region has experienced uneven economic growth, so we assume that RHD is unequally distributed between countries in the Region [16,17]. The aim of this study was to comprehensively assess the burden, trends, and health inequalities of RHD in the Asian Region by conducting a secondary analysis of the global burden of disease. The intention was to provide evidence to inform policymakers on how to allocate healthcare resources more effectively, promote healthcare equity, and reduce health disparities between countries.

## Methods

### Data sources

The data for this study were obtained from the Global Health Data Exchange GBD Results Tool (https://vizhub.healthdata.org/gbd-results/), which provided a systematic assessment of age- and sex-specific mortality for 282 causes, prevalence, and years lived with disability for 369 diseases and injuries, and comparative risks for more than 80 risk factors in 204 countries and territories, from 1 January 1990, to 31 December 2019 [18–22]. To assess the burden of RHD in the Asian Region, we extracted estimates and 95% uncertainty intervals (UI) for the number of cases, deaths, age-standardised prevalence, mortality, disability-adjusted life years (DALYs), disability-loss healthy life years (YLDs), and life loss years (YLLs) for global, Asian, Asian sub-regions, and national RHD from 1990–2019. RHD (ICD-10 codes: I01-I01.9, I02.0, and I05-I09.9) was defined as a clinical diagnosis by a physician with or without confirmation using echocardiography.

Gross Domestic Product (GDP) per capita estimates from the Institute for Health Metrics and Evaluation for 1990 and 2019 (in 2020 PPP-adjusted dollars) were obtained from the Global Burden of Disease Collaborative Network [19].

### GBD estimation framework

An integrated cause of death model (CODEm) approach was used to model deaths due to RHD based on vital sign registration and monitoring data [20]. The covariates used in the modelling process are shown in Supplementary Table S6. The basic tabular listing data points for ICD8 and ICD9 were dropped from the analysis because they were inconsistent with the rest of the data and produced implausible time trends. Data points too high after re-assignment in some age groups were similarly excluded.

The non-fatal disease burden of RHD was modelled using the DisMod-MR 2.1 tool to obtain incidence and prevalence rates using hospital data, claims data, and prevalence literature data [21]. DisMod-MR 2.1 is a Bayesian meta-regression disease modelling tool. The detailed methodology of GBD 2019 has been described elsewhere [22,23].

Disability-adjusted life years (DALYs) were calculated as the sum of years of life lost (YLLs) and years of disability life (YLDs). RHD-related YLLs were calculated by multiplying the number of deaths in each age group by the standard life expectancy for that age group. YLDs were calculated by multiplying the prevalence of each mutually exclusive sequelae by its disability weight.

### Data analysis

We assessed the burden of RHD in the Asian Region by conducting a secondary analysis of GBD 2019 data. We used descriptive analysis to describe the burden of RHD in the Asian Region. The number of cases, deaths, and age-standardised prevalence, mortality, DALYs, YLDs, and YLLs (per 100 000 population) were compared for both sexes combined. We also explored the burden of RHD in 48 Asian countries and territories. Statistical analysis and graphing for this study were done by R (software version 4.1.1) software.

We used the Joinpoint regression programme (software version 4.9.1.0.) to obtain the average annual percentage change (AAPC) in mortality, prevalence, DALYs, YLDs, and YLLs for RHD from 1990 to 2019 and their 95% confidence intervals (CI) as a way to assess the 1990–2019 RHD trends in the burden of RHD. We also segmented by age group (5–14 years, 15–49 years, 50–69 years, 70 years and over, and all ages [age-standardised]) at the global, regional, sub-regional, and country levels. AAPC is a summary measure of trends within a pre-specified fixed interval, a weighted average of the annual percentage change calculated by the Joinpoint model, with a weight equal to the length of the annual percentage change interval. AAPC was considered significant when it differed from zero at the alpha (two-sided) level of 0.05. The trend is considered constant when the zero value is within the 95% CI of the AAPC. When both bounds of the 95% CI are positive, it is an uptrend, and when both bounds of the 95% CI are negative, it is a downtrend. The statistical analysis for this study was conducted in 2022.

### Country-level inequalities analysis

Health inequities are the unjust differences in health between people of different social groups and can be linked to forms of disadvantages such as poverty, discrimination, and a lack of access to services or goods [24]. The inequality distribution of the RHD burden across countries and territories was measured by the slope index of inequality (SII) and the relative index of inequality (RII), corresponding to absolute and relative inequality, respectively [24]. We calculated the inequality slope index by regressing the age-standardised mortality rate of RHD on the relative social status rank associated with income for all age populations at the country level. The rank is the midpoint of the cumulative population distribution for each category after ranking the entire population by GDP per capita. The difference between the highest and lowest values of the rank generates the inequality slope index, representing the difference between the lowest and highest values. Since this ranking is weighted, all other subgroups are also considered in the regression (the effect of change in the whole population distribution by wealth). When the inequality slope index is positive, mortality is more prevalent in the highest-status subgroup. At the same time, a negative value means the indicator is more prevalent in the lowest-status subgroup. There is no difference in mortality when the inequality slope index is zero. The relative index of inequality was calculated by dividing the inequality slope index by the mean age-standardised mortality rate for RHD in the population of all ages. This index represents the ratio of the age-standardised mortality rate of the lowest social status subgroup relative to the mean of the entire population. When it is one, it indicates that the age-standardised mortality rates of the two groups are equivalent. And we also compared age-standardised mortality rates in 1990 and 2019 across different income groups to assess how they changed.

### Classification of countries based on YLLs reduction

**Figure 1.**
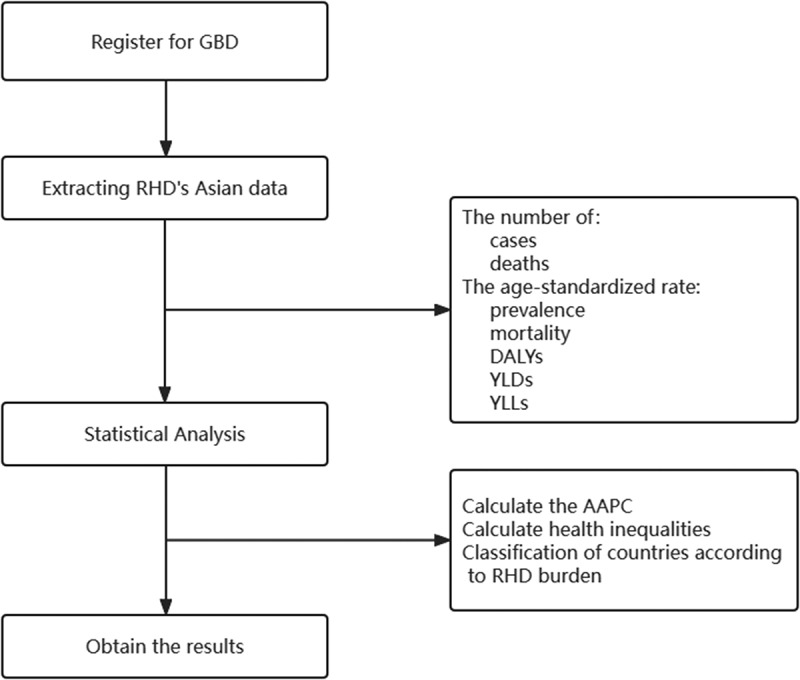
Study flow chart.

We divided Asian countries and territories into nine categories by calculating the 33rd and 66th percentiles (lower and upper terciles) of the AAPC of the age-standardised YLLs rate (per 100,000 population) from 1990 to 2019 and the age-standardised YLLs rate (per 100,000 population) in 2019. See the flow chart in Figure 1.

## Results

According to GBD 2019, there were an estimated 22 246 127 (95% UI 17 529 847 to 27 743 618) RHD cases and 249 830 deaths (202 631 to 284 553) in the Asian all-age population in 2019. The age-standardised prevalence in the Asian Region was 9.4% lower (465.6 cases per 100 000 population [95% UI 366.9 to 580.1] vs. 513.7 cases per 100 000 population [405.0 to 636.3]) than the global estimate, whereas the age-standardised mortality rate was 41.0% higher (5.5 deaths per 100 000 population [4.5 to 6.3] vs 3.9 deaths per 100 000 population [3.3 to 4.3]). Age-standardised mortality rates trended downwards in both the Asian Region (AAPC: −3.2%, 95% UI −3.3 to −3.1) and globally (AAPC: −2.9%, 95% UI −3.0 to −2.8) between 1990 and 2019. Detailed data are summarised in Table 1.

**Table 1.** Global and Asian prevalence, mortality, and burden of rheumatic heart disease in 2019 and their trends from 1990–2019.

	Global	Asia
**2019**		
Prevalence		
Number of cases 2019	40 502 345 (32 052 904 to 50 062 426)	22 246 127 (17 529 847 to 27 743 618)
Age-standardized prevalence 2019 (per 100 000 population)	513.7 (405.0 to 636.3)	465.6 (366.9 to 580.1)
Mortality		
Number of deaths 2019	305 651 (259 220 to 340 486)	249 830 (202 631 to 284 553)
Age-standardized mortality 2019 (per 100 000 population)	3.9 (3.3 to 4.3)	5.5 (4.5 to 6.3)
Burden of disease		
Age-standardized DALYs (per 100 000 population)	132.9 (115.0 to 150.3)	175.2 (146.3 to 199.5)
Age-standardized YLLs (per 100 000 population)	107.7 (92.7 to 120.9);81% of DALYs	152.0 (125.4 to 173.3);87% of DALYs
Age-standardized YLDs (per 100 000 population)	25.2 (15.2 to 38.7);19% of DALYs	23.2 (13.9 to 35.4);13% of DALYs
**1990–2019**		
Prevalence (age-standardized)		
AAPC from 1990–2019	0.5% (0.4 to 0.5)	0.1% (0.1 to 0.2)
AAPC from 1990–1999	−0.1% (−0.1 to 0)	−0.5% (−0.5 to −0.5)
AAPC from 2000–2009	0.8% (0.8 to 0.8)	0.7% (0.6 to 0.7)
AAPC from 2010–2019	0.6% (0.5 to 0.7)	0.3% (0.2 to 0.3)
Mortality (age-standardized)		
AAPC from 1990–2019	−2.9% (−3.0 to −2.8)	−3.2% (−3.3 to −3.1)
AAPC from 1990–1999	−2.7% (−3.0 to −2.4)	−3.0% (−3.3 to −3.1)
AAPC from 2000–2009	−3.3% (−3.4 to −3.2)	−3.6% (−3.7 to −3.4)
AAPC from 2010–2019	−2.6% (−2.8 to −2.5)	−3.1% (−3.2 to −3.0)

**Figure 2.**
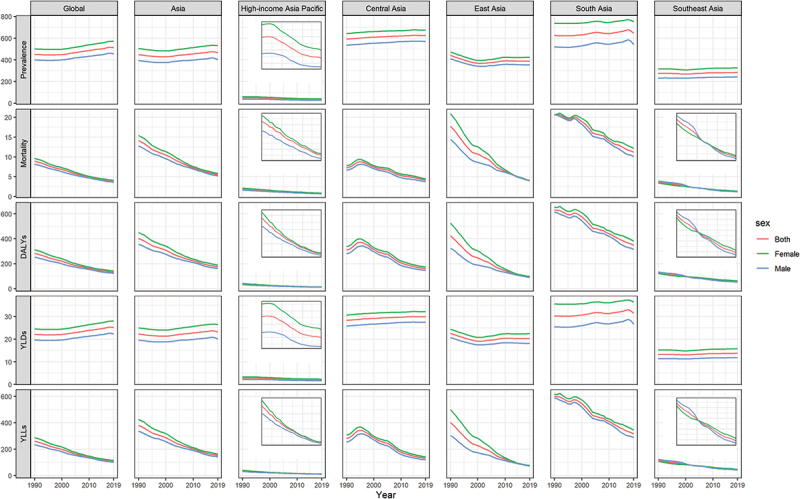
Rheumatic heart disease prevalence, mortality, DALYs, YLDs, and YLLs per 100 000 population by sex at global, regional, and subregional levels, 1990–2019.

RHD age-standardised prevalence and YLDs in the Asian Region and its sub-regions (except high-income Asia-Pacific) did not change or increase slightly between 1990 and 2019, mirroring a global trend (Figure 2). RHD-related age-standardised mortality rates and YLLs were higher in the South Asian sub-region than in the Asian Region, similar to the Asian Region in the Central and West Asian sub-regions, and much lower in the Southeast Asian and high-income Asia-Pacific sub-regions. Still, all sub-regions showed a decreasing trend between 1990 and 2019 (Figure 2). Age-standardised prevalence and mortality rates were generally higher in women than men, but the mortality gap by sex was reduced (Figure 2).

From 1990 to 2019, most countries showed a significant decline in mortality, with an AAPC of −2.3% (95% UI −3.3 to −1.4) for the whole region, ranging from −7.8% in Qatar (95% UI −9.3 to −6.3) and Singapore (95% UI −9.2 to −6.4) to 2.0% in the Philippines (95% UI 1.3 to 2.7). The decreasing trend in mortality in most countries and territories was greater than that observed in the Asian Region (Table 2).

**Table 2.** Changes in rheumatic heart disease deaths at age 5–14 years and age-standardised YLLs at all ages, 1990–2019.

	Mortality at 5–14 years of age		YLLs at all ages	
	Deaths per 100 000 population (95% UI), 2019	Average annual percentage change (95% CI), 1990–2019	Age-standardized YLLs per 100 000 population (95% UI), 2019	Average annual percentage change (95% CI), 1990–2019
Global	0.33 (0.27 to 0.38)	−2.6% (−3.3 to −1.9)	107.67 (92.66 to 120.94)	−3.0% (−3.2 to −2.9)
Asia	0.42 (0.34 to 0.49)	−2.3% (−3.3 to −1.4)	151.96 (125.44 to 173.31)	−3.1% (−3.3 to −3.0)
Central Asia	0.17 (0.14 to 0.21)	−3.4% (−4.1 to −2.6)	131.36 (112.63 to 151.17)	−2.6% (−2.9 to −2.3)
East Asia	0.05 (0.04 to 0.06)	−7.4% (−8.2 to −6.6)	73.88 (61.34 to 85.97)	−5.7% (−5.9 to −5.4)
High-income Asia Pacific	0.01 (0.01 to 0.01)	−5.3% (−5.8 to −4.9)	11.20 (9.66 to 12.22)	−3.9% (−4.1 to −3.7)
South Asia	0.64 (0.50 to 0.78)	−2.3% (−3.6 to −0.9)	316.97 (243.57 to 380.96)	−2.2% (−2.5 to −1.9)
Southeast Asia	0.34 (0.29 to 0.38)	−1.6% (−2.1 to −1.1)	44.71 (38.81 to 51.01)	−3.2% (−3.4 to −3.0)
Afghanistan	0.37 (0.21 to 0.65)	−3.6% (−4.1 to −3.2)	186.52 (118.01 to 277.88)	−2.4% (−2.5 to −2.2)
Armenia	0.04 (0.02 to 0.06)	−4.2% (−5.1 to −3.2)	72.54 (56.28 to 91.13)	−3.9% (−4.5 to −3.3)
Azerbaijan	0.15 (0.09 to 0.22)	−2.5% (−3.2 to −1.9)	60.48 (45.85 to 78.38)	−2.9% (−3.4 to −2.5)
Bahrain	0.07 (0.05 to 0.10)	−4.4% (−6.1 to −2.7)	23.64 (18.68 to 30.18)	−3.2% (−3.9 to −2.5)
Bangladesh	0.48 (0.31 to 0.71)	−1.5% (−2.3 to −0.6)	78.69 (58.54 to 100.30)	−2.2% (−3.3 to −1.1)
Bhutan	0.44 (0.26 to 0.68)	−1.3% (−1.7 to −0.9)	310.50 (155.48 to 611.28)	−2.8% (−2.9 to −2.7)
Brunei Darussalam	0.06 (0.04 to 0.08)	−4.1% (−5.0 to −3.2)	54.67 (46.45 to 63.38)	−2.4% (−2.5 to −2.3)
Cambodia	0.34 (0.22 to 0.48)	−4.2% (−4.4 to −4.1)	98.97 (63.36 to 140.39)	−3.8% (−4.0 to −3.6)
China	0.05 (0.04 to 0.06)	−7.5% (−8.4 to −6.7)	73.33 (60.04 to 85.94)	−5.8% (−6.0 to −5.5)
Cyprus	0.02 (0.01 to 0.03)	−3.6% (−4.8 to −2.4)	39.34 (33.43 to 47.09)	−3.9% (−4.2 to −3.5)
Democratic People’s Republic of Korea	0.11 (0.06 to 0.21)	−2.9% (−3.2 to −2.6)	185.64 (114.50 to 277.14)	−1.6% (−1.7 to −1.5)
Georgia	0.54 (0.40 to 0.71)	−2.0% (−4.0 to 0.0)	145.27 (117.27 to 175.60)	0.1% (−0.4 to 0.5)
India	0.49 (0.38 to 0.62)	−3.4% (−5.5 to −1.2)	319.42 (244.66 to 390.49)	−2.4% (−2.8 to −2.0)
Indonesia	0.12 (0.09 to 0.16)	−4.7% (−4.9 to −4.5)	18.79 (16.08 to 21.97)	−4.5% (−4.7 to −4.4)
Iran (Islamic Republic of)	0.18 (0.15 to 0.21)	−5.1% (−5.6 to −4.6)	36.67 (32.18 to 41.48)	−3.7% (−4.1 to −3.4)
Iraq	0.07 (0.05 to 0.11)	−7.4% (−8.0 to −6.9)	39.34 (29.98 to 50.88)	−4.8% (−5.0 to −4.6)
Israel	0.03 (0.02 to 0.04)	−4.2% (−5.0 to −3.5)	31.00 (26.28 to 36.65)	−2.4% (−3.4 to −1.5)
Japan	0.01 (0.01 to 0.01)	−2.9% (−3.4 to −2.4)	11.94 (10.20 to 13.13)	−3.6% (−4.0 to −3.3)
Jordan	0.04 (0.02 to 0.06)	−4.7% (−5.4 to −3.9)	11.40 (9.04 to 14.12)	−4.6% (−4.9 to −4.3)
Kazakhstan	0.04 (0.03 to 0.06)	−5.8% (−7.0 to −4.6)	63.08 (47.14 to 81.40)	−5.0% (−5.4 to −4.6)
Kuwait	0.06 (0.04 to 0.09)	−6.5% (−7.8 to −5.2)	10.35 (8.10 to 13.27)	−5.4% (−6.4 to −4.5)
Kyrgyzstan	0.12 (0.09 to 0.16)	−4.5% (−6.1 to −3.0)	113.90 (89.45 to 142.44)	−4.0% (−4.8 to −3.2)
Lao People’s Democratic Republic	0.96 (0.61 to 1.41)	−2.2% (−2.5 to −1.9)	139.54 (95.87 to 189.13)	−2.6% (−2.8 to −2.5)
Lebanon	0.07 (0.03 to 0.14)	−5.7% (−5.9 to −5.5)	22.74 (10.76 to 34.54)	−4.2% (−4.3 to −4.1)
Malaysia	0.14 (0.09 to 0.21)	−6.0% (−7.1 to −5.0)	26.37 (19.16 to 34.25)	−5.6% (−5.9 to −5.4)
Maldives	0.21 (0.07 to 0.34)	−6.9% (−8.0 to −5.8)	32.04 (24.78 to 39.40)	−6.2% (−6.5 to −5.9)
Mongolia	0.11 (0.06 to 0.18)	−3.0% (−4.0 to −1.9)	139.43 (105.19 to 181.22)	−3.2% (−3.3 to −3.1)
Myanmar	0.23 (0.14 to 0.37)	−4.7% (−4.8 to −4.5)	75.53 (54.41 to 103.31)	−3.6% (−3.7 to −3.5)
Nepal	0.35 (0.21 to 0.52)	−3.4% (−3.7 to −3.2)	332.87 (215.86 to 484.76)	−2.6% (−2.7 to −2.5)
Oman	0.03 (0.02 to 0.03)	−5.2% (−5.8 to −4.6)	11.31 (9.32 to 13.59)	−4.6% (−4.9 to −4.3)
Pakistan	1.43 (0.94 to 1.96)	0.8% (0.4 to 1.2)	504.35 (371.13 to 656.07)	−1.2% (−1.3 to −1.1)
Palestine	0.02 (0.01 to 0.03)	−6.3% (−7.7 to −4.9)	16.61 (13.36 to 19.88)	−4.2% (−4.4 to −4.1)
Philippines	1.09 (0.85 to 1.26)	2.0% (1.3 to 2.7)	107.52 (83.32 to 128.98)	1.5% (1.0 to 1.9)
Qatar	0.04 (0.02 to 0.07)	−7.8% (−9.3 to −6.3)	15.97 (12.05 to 20.94)	−5.2% (−5.5 to −4.9)
Republic of Korea	0.01 (0.01 to 0.01)	−7.6% (−8.3 to −6.9)	6.80 (5.73 to 7.98)	−4.8% (−4.9 to −4.7)
Saudi Arabia	0.04 (0.02 to 0.06)	−7.1% (−7.4 to −6.8)	27.74 (19.07 to 37.47)	−4.3% (−4.7 to −4.0)
Singapore	0.02 (0.01 to 0.03)	−7.8% (−9.2 to −6.4)	8.56 (7.01 to 10.53)	−6.9% (−7.2 to −6.7)
Sri Lanka	0.08 (0.04 to 0.14)	−4.2% (−8.2 to 0.0)	22.39 (15.54 to 30.67)	−4.7% (−5.3 to −4.2)
Syrian Arab Republic	0.49 (0.31 to 0.71)	−6.0% (−7.0 to −5.1)	52.75 (36.65 to 72.44)	−6.5% (−7.1 to −6.0)
Tajikistan	0.14 (0.09 to 0.20)	−3.6% (−4.3 to −3.0)	121.46 (96.72 to 153.13)	−3.5% (−3.9 to −3.1)
Thailand	0.14 (0.10 to 0.21)	−4.5% (−5.3 to −3.7)	15.47 (11.62 to 20.15)	−7.5% (−8.1 to −6.9)
Timor-Leste	0.68 (0.40 to 1.04)	−0.7% (−1.1 to −0.3)	108.65 (64.19 to 159.84)	−1.7% (−2.2 to −1.2)
Turkey	0.02 (0.01 to 0.03)	−4.0% (−4.9 to −3.0)	13.62 (10.72 to 16.98)	−4.1% (−4.4 to −3.9)
Turkmenistan	0.18 (0.12 to 0.26)	−3.5% (−4.7 to −2.4)	95.44 (70.02 to 126.38)	−3.1% (−4.0 to −2.2)
United Arab Emirates	0.17 (0.09 to 0.29)	−5.6% (−5.9 to −5.3)	83.90 (49.87 to 128.94)	−3.4% (−3.8 to −3.1)
Uzbekistan	0.25 (0.18 to 0.33)	−3.2% (−4.2 to −2.3)	204.61 (157.56 to 256.65)	−1.9% (−2.4 to −1.4)
Viet Nam	0.06 (0.04 to 0.09)	−3.8% (−4.0 to −3.7)	41.41 (28.74 to 53.66)	−3.8% (−3.9 to −3.7)
Yemen	0.55 (0.32 to 0.89)	−2.8% (−3.2 to −2.4)	117.45 (74.12 to 181.96)	−2.6% (−2.8 to −2.4)

**Figure 3.**
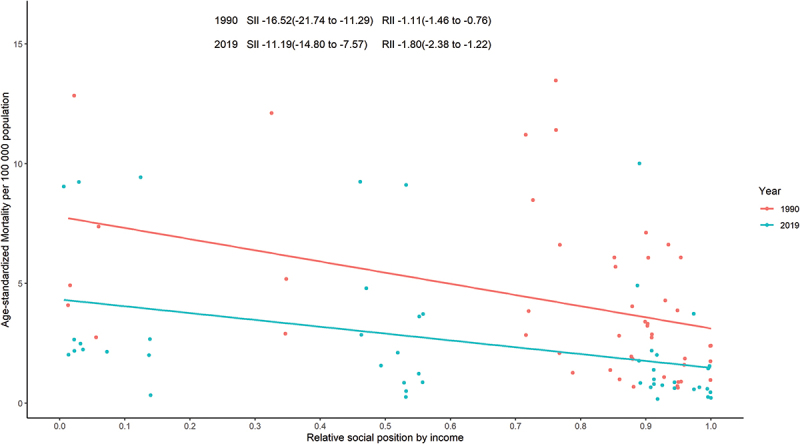
Age-standardized YLLs per 100 000 in 2019 and its AAPC of RHD for Asian 48 countries and territories. Age-standardized YLLs of RHD per 100 000 population from 1990 through 2019 stratified by region (A) or sex (C). Age-standardized YLLs of RHD per 100 000 population by country and territory, in 2019 (B). The relative changes in age-standardized YLLs of RHD by country and territory from 1990 through 2019 (D). RHD: rheumatic heart disease.

The age-standardised YLLs for all ages in the Asian Region were 151.96 per 100 000 (95% UI 125.44 to 173.31), with a wide variation across countries and territories (from 6.80 per 100 000 [95% UI 5.73 to 7.98] in Korea to 504.35 per 100 000 [95% UI 371.13 to 656.07] in Pakistan), and seven countries having higher YLLs than the Asian Region (Table 2; Figure 3). The majority of countries showed a decreasing trend in age-standardised YLLs between 1990 and 2019, with 31 out of 48 countries and territories showing a decreasing trend above the Asia Region, while the Philippines showed an increasing trend in YLLs (1.5%, 95% UI 1.0 to 1.9) (Table 2; Figure 3).

Age-standardised mortality rates decreased significantly for each age group during 1990–2019, with the largest decrease in the 50–69 age group (Appendix Table A2). The total number of deaths and YLLs per 100 000 population in 2019, as well as the AAPC and 95% CI from 1990 to 2019 for mortality and YLLs by age group (5–14 years, 15–49 years, 50–69 years, and 70 years and older) for both sexes combined, were computed and are available in Appendix Table A2. Globally and also in the Asian Region, those over 70 years of age have the largest share of the burden of death at 44.6% and 42.0%, respectively, with details available in the Appendix, Figure A6.

**Figure 4.**
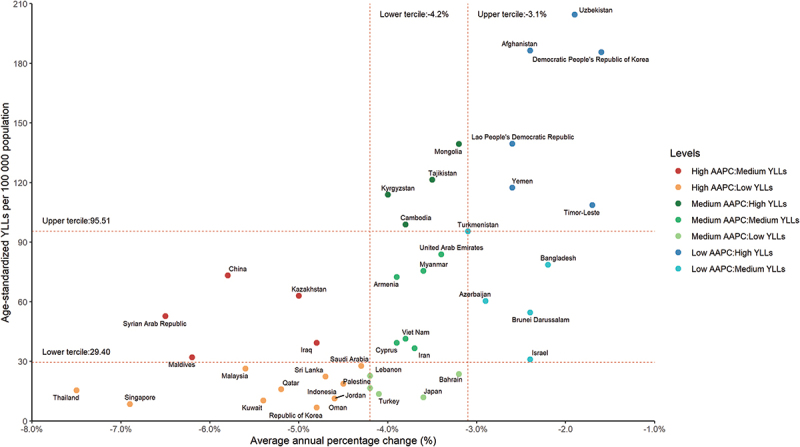
Income-related health inequality regression curves for the mortality due to rheumatic heart disease across Asia, 1990 and 2019. SII: slope index of inequality; RII: relative index of inequality.

Among the 48 Asian countries and territories, there are clear absolute and relative inequalities in the distribution of RHD-related mortality, with this burden disproportionately concentrated in poorer countries. From 1990 to 2019, absolute inequality between countries and territories decreased while relative inequality increased. As the slope index of inequality shows, the mortality rate in the poorest quintile was 16.52 per 100 000 population (11.29 to 21.74), higher than in the richest quintile in 1990, with the gap falling to 11.19 per 100 000 population (7.57 to 14.80) in 2019 (Figure 4). Regional relative inequality was measured using the relative index of inequality, which in 1990 was −1.11 (−1.46 to −0.76), indicating that RHD-related mortality in the poorest quintile was 11% higher than the regional average. The relative index of inequality for 2019 was −1.80 (−2.38 to −1.22), meaning that the poorest quintile has a mortality rate of 80% above the average (Figure 4). From 1990 to 2019, mortality rates in the poorest quintile (below the Asian average) fell by a factor of one, while mortality rates in the richest quintile fell by a factor of two, while mortality rates in the intermediate groups remained essentially unchanged (Appendix Table A1; Appendix Figure A2).

**Figure 5.**
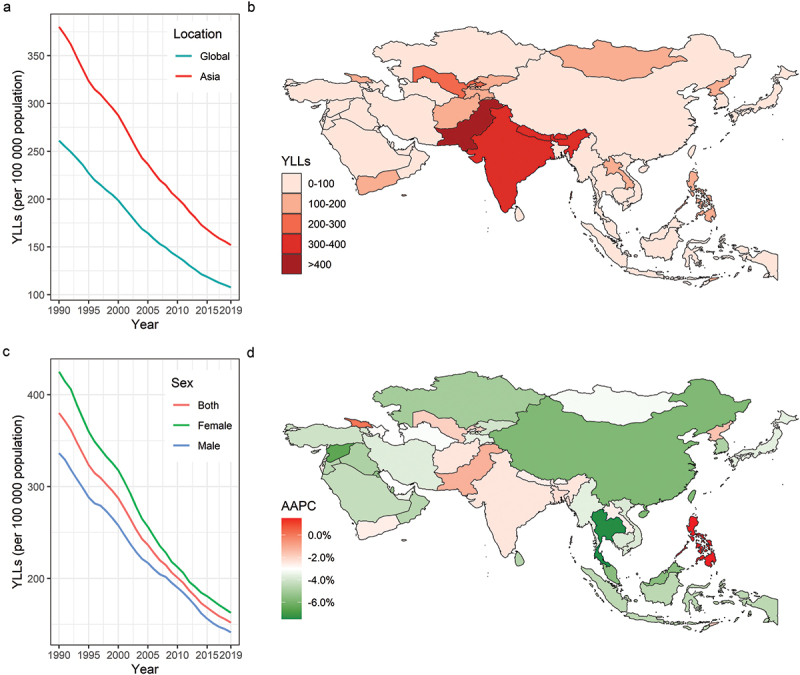
Age-standardized YLLs per 100 000 in 2019 and AAPC in age-standardized YLLs per 100 000 in 1990–2019 due to rheumatic heart disease for all ages and both sexes in Asia.

In the Asian Region, 16 of the 48 countries and territories (33%), including Bhutan, India, Nepal, and Pakistan (not shown on the scatter plot because their YLLs exceed 300 per 100,000), had YLLs in 2019 in the upper tercile of the distribution (Figure 5), which represents the countries and territories with the highest number of lost life years in 2019. In addition, 12 of these 16 countries were in the upper tier of the AAPC distribution from 1990 to 2019, representing the group with the smallest reduction in YLLs: Afghanistan, Bhutan, the Democratic People’s Republic of Korea, Georgia (not shown on the scatter chart because their AAPC exceeds 0%), India, Lao People’s Democratic Republic, Nepal, Pakistan, the Philippines (not shown on the scatter plot because its AAPC exceeds 0%), Timor-Leste, Uzbekistan, and Yemen. Countries and territories located in the lower tier of the distribution were those with the largest reductions in lost life years and mortality.

## Discussion

The vast majority of the published literature studying the burden of RHD has described overall trends in a global context. Little attention has been paid to RHD in the Asian Region. In addition, studies have shown that aggregated national and regional-level data do not fully describe the burden within sub-populations; country-specific information needs to be collected and used [25]. Our secondary analysis of GBD 2019 data provided an up-to-date description of the epidemiology of RHD in 48 countries and territories in the Asian Region. This study improves understanding of RHD governance patterns in the Asian Region and identifies countries and territories that need to strengthen prevention and control. Our findings showed a significant decreasing trend in age-standardised mortality rates, DALYs, and YLLs for RHD in the Asian Region from 1990 to 2019. This may be related to the great achievements of the Asian Region in the economic field. Asia’s real GDP in purchasing power parity (PPP) terms climbed from about $3.3 trillion in 1980 to an estimated $24.5 trillion in 2009, which was an increase of 7.5 times [16]. Asia’s economic growth has led to improved medical facilities, which have reduced RHD mortality, DALYs, and YLLs. In addition, policies in countries such as India, Bangladesh, and Nepal for preventing and controlling non-communicable diseases may also contribute to the declining burden of RHD [26]. Strategies have reduced the exposure of the population to risk factors. We also found that the prevalence of RHD in the Asian Region was slightly lower than the global level, but its mortality rate was much higher than the global level. Many factors influence RHD mortality, such as timely detection by echocardiography, widespread use of penicillin, and good surgical care. All of the above are associated with economic development and the construction and improvement of healthcare systems nationally and at regional levels [2]. In the last 30 years, Asia has made great progress in economic construction and public health [16]. However, Asian countries have been lagging in economic and healthcare measures (Asian GDP per capita vs. World GDP per capita: 1/4 in 1980, 2/3 in 2019), and this may be responsible for the much higher RHD mortality in the Asian Region compared with the global average [17,27]. Our findings indicated that while RHD governance in the Asian Region has improved significantly, it still lags behind the global average and remains a serious public health issue that requires more attention.

Notably, our findings showed a higher burden of RHD in women than in men, which was consistent with some previous studies [28–30]. This may be due to the lower education level of girls in less economically developed areas or the higher cumulative exposure to β-hemolytic streptococci in females compared to males [31]. Meanwhile, several studies have highlighted RHD’s high morbidity and mortality during pregnancy, which may seriously affect fertility quality [32,33]. Therefore, to improve the global population’s health, more attention must be paid to the particular burden of RHD on women of childbearing age, especially in areas with poor healthcare and cardiac evaluation and treatment of these high-risk women before and after during pregnancy is recommended.

Our analysis of RHD health inequalities in the Asian Region showed a clear gradient in RHD burden between countries and territories ranked by level of GDP per capita (the higher the GDP per capita, the lower the burden). Among the many social determinants that influence population health, wealth or income is considered a central factor. Its distributional inequality impacts health by influencing access to health care and health outcomes [14,34–36]. As a region with extremely uneven development, the existence of health inequalities in the Asian Region was not surprising [17].

We discovered that while the gap between RHD mortality in the poorest and richest quintiles has narrowed over time (absolute inequality has decreased), the proportion of mortality exceeding the Asian average in the poorest quintile has increased (relative inequality has increased). We found that in the last 30 years, mortality rates in the poorest quintile decreased by one factor and two in the richest quintile, while mortality rates in the intermediate groups remained unchanged. The mortality rate in the poorest quintile was much higher in 1990 than in the richest quintile.

While the death rate in the former only decreased by a factor of one between 1990 and 2019, the decline was much greater than in the richest quintile of countries and territories. Our results showed that Asia had achieved a lot in the overall governance of RHD in the last 30 years, but that the region was unbalanced in the distribution of healthcare resources. There is an urgent need to focus more attention on the governance of poor and middle-income countries. In particular, the COVID-19 pandemic has further increased inequalities in most parts of the world [37]. Our classification of 48 Asian countries and territories based on YLLs and their changing trends provided a more nuanced analysis. The study shows that RHD remains a significant public health problem and provides evidence to enable policymakers to allocate healthcare resources better.

Our study has several limitations. First, GBD did not include RHD subclinical cases in the model when estimating the prevalence of RHD, which would have resulted in a lower estimated prevalence than the actual value. Secondly, most of the raw data used for GBD estimation comes from developed countries and territories, and the data obtained from low- and middle-income countries was not of high quality, which could cause deviations between the estimated and actual values. Thirdly, when the linkage point approach was used to estimate AAPC, the data was derived from GBD rather than raw data for each region, which may lead to an underestimation of the uncertainty in AAPC trends. Finally, because of the Asian Region’s geographic and economic diversity, between-country comparisons of RHD are not always clear-cut. Despite these limitations, the GBD 2019 data are still extremely useful for policymakers to implement RHD prevention interventions and inform policy settings.

## Conclusions

Although the burden of RHD in the Asian Region has continued to decrease, it remains a serious public health problem that requires urgent policy attention. At the same time, inequalities in the distribution of the RHD burden in the Asian Region remain significant, with the burden generally higher in economically disadvantaged areas. Policymakers can use GBD 2019 data to allocate healthcare resources better and reduce inequalities in the distribution of RHD burden in the Asian Region.

## Supplementary Material

Supplemental Material

## Paper context

Evidence of the burden of Rheumatic Heart Disease and health inequalities between countries and territories can be used to improve the allocation of health resources and reduce health disparities between countries. This paper examines the burden of Rheumatic Heart Disease and health inequalities in 48 countries and territories in the Asian Region. The findings show that Rheumatic Heart Disease remains a very serious public health problem and that significant health disparities in this Disease exist between countries.
