# Supplementary material for: Rheumatic heart disease burden, trends, and inequalities in Asia, 1990–2019
Source: Glob Health Action. 2023 May 26;16(1):2215011. doi: 10.1080/16549716.2023.2215011 (PMC10228324; doi:10.1080/16549716.2023.2215011)
Supplement: Supplemental Material [file ZGHA_A_2215011_SM3286.docx]

Appendix table 1: Age-standardized mortality and their changes in different income groups

|  | Age-standardized mortality per 100 000 population | | Percentage of decline |
| --- | --- | --- | --- |
|  | 1990 | 2019 |  |
| The poorest quintile | 18.38 | 9.02 | 104% |
| The intermediate groups | 6.71 | 6.11 | 10% |
| The richest quintile | 2.82 | 0.86 | 226% |
| Average | 14.89 | 6.21 | 140% |

Appendix table 2: Rheumatic heart disease death rates per 100 000 population in 2019 and its average annual percentage change in the period 1990-2019, and age-standardized years of life lost rates per 100 000 population in 2019 and its average annual percentage change in the period 1990-2019 by age group, both sexes combined, and location

|  | **5-14 years** |  |  |  |  |  |
| --- | --- | --- | --- | --- | --- | --- |
|  | Death | | | Years of life lost (YLLs) | | |
| Location Name | rate per 100 000 population, 2019 | Average annual percent change (95% CI), 1990-2019 | P-value | rates per 100 000 population, 2019 | Average annual percent change (95% CI),1990-2019 | P-value |
| Global | 0.33 (0.27 to 0.38) | -2.6% (-3.3 to -1.9) | <0.001 | 23.52 (21.22 to 29.96) | -2.6% (-3.3 to -1.9) | <0.001 |
| Asia | 0.42 (0.34 to 0.49) | -2.3% (-3.3 to -1.4) | <0.001 | 32.70 (26.62 to 38.61) | -2.4% (-3.3 to -1.4) | <0.001 |
| Central Asia | 0.17 (0.14 to 0.21) | -3.4% (-4.1 to -2.6) | <0.001 | 13.48 (10.87 to 16.37) | -3.4% (-4.1 to -2.7) | <0.001 |
| East Asia | 0.05 (0.04 to 0.06) | -7.4% (-8.2 to -6.6) | <0.001 | 3.75 (3.15 to 4.54) | -7.4% (-8.3 to -6.6) | <0.001 |
| High-income Asia Pacific | 0.01 (0.01 to 0.01) | -5.3% (-5.8 to -4.9) | <0.001 | 0.83 (0.65 to 0.95) | -5.3% (-5.7 to -4.9) | <0.001 |
| South Asia | 0.64 (0.50 to 0.78) | -2.3% (-3.6 to -0.9) | 0.001 | 50.04 (39.32 to 60.71) | -2.3% (-3.7 to -0.9) | 0.001 |
| Southeast Asia | 0.34 (0.29 to 0.38) | -1.6% (-2.1 to -1.1) | <0.001 | 26.31 (22.46 to 30.12) | -1.6% (-2.2 to -1.1) | <0.001 |
| Afghanistan | 0.37 (0.21 to 0.65) | -3.6% (-4.1 to -3.2) | <0.001 | 29.27 (16.68 to 51.52) | -3.6% (-4.1 to -3.2) | <0.001 |
| Armenia | 0.04 (0.02 to 0.06) | -4.2% (-5.1 to -3.2) | <0.001 | 3.27 (1.85 to 4.50) | -4.2% (-5.1 to -3.2) | <0.001 |
| Azerbaijan | 0.15 (0.09 to 0.22) | -2.5% (-3.2 to -1.9) | <0.001 | 11.39 (7.25 to 17.03) | -2.5% (-3.2 to -1.9) | <0.001 |
| Bahrain | 0.07 (0.05 to 0.10) | -4.4% (-6.1 to -2.7) | <0.001 | 5.39 (3.63 to 7.64) | -4.4% (-6.1 to -2.7) | <0.001 |
| Bangladesh | 0.48 (0.31 to 0.71) | -1.5% (-2.3 to -0.6) | 0.001 | 37.52 (23.73 to 54.65) | -1.5% (-2.3 to -0.6) | 0.001 |
| Bhutan | 0.44 (0.26 to 0.68) | -1.3% (-1.7 to -0.9) | <0.001 | 34.14 (20.41 to 52.27) | -1.4% (-1.8 to -1.0) | <0.001 |
| Brunei Darussalam | 0.06 (0.04 to 0.08) | -4.1% (-5.0 to -3.2) | <0.001 | 4.40 (2.87 to 6.58) | -4.1% (-5.0 to -3.2) | <0.001 |
| Cambodia | 0.34 (0.22 to 0.48) | -4.2% (-4.4 to -4.1) | <0.001 | 27.02 (17.50 to 37.79) | -4.3% (-4.4 to -4.1) | <0.001 |
| China | 0.05 (0.04 to 0.06) | -7.5% (-8.4 to -6.7) | <0.001 | 3.67 (3.07 to 4.52) | -7.6% (-8.4 to -6.7) | <0.001 |
| Cyprus | 0.02 (0.01 to 0.03) | -3.6% (-4.8 to -2.4) | <0.001 | 1.66 (1.05 to 2.52) | -3.6% (-4.8 to -2.4) | <0.001 |
| Democratic People's Republic of Korea | 0.11 (0.06 to 0.21) | -2.9% (-3.2 to -2.6) | <0.001 | 8.87 (4.85 to 16.07) | -2.9% (-3.2 to -2.6) | <0.001 |
| Georgia | 0.54 (0.40 to 0.71) | -2.0% (-4.0 to 0.0) | 0.051 | 42.29 (31.75 to 55.42) | -2.0% (-4.0 to 0.0) | 0.049 |
| India | 0.49 (0.38 to 0.62) | -3.4% (-5.5 to -1.2) | 0.002 | 38.50 (29.40 to 48.71) | -3.4% (-5.5 to -1.3) | 0.002 |
| Indonesia | 0.12 (0.09 to 0.16) | -4.7% (-4.9 to -4.5) | <0.001 | 9.24 (7.26 to 12.20) | -4.7% (-4.9 to -4.5) | <0.001 |
| Iran (Islamic Republic of) | 0.18 (0.15 to 0.21) | -5.1% (-5.6 to -4.6) | <0.001 | 13.93 (11.62 to 16.38) | -5.2% (-5.7 to -4.6) | <0.001 |
| Iraq | 0.07 (0.05 to 0.11) | -7.4% (-8.0 to -6.9) | <0.001 | 5.84 (3.94 to 8.50) | -7.5% (-8.0 to -6.9) | <0.001 |
| Israel | 0.03 (0.02 to 0.04) | -4.2% (-5.0 to -3.5) | <0.001 | 2.06 (1.38 to 2.84) | -4.3% (-5.0 to -3.5) | <0.001 |
| Japan | 0.01 (0.01 to 0.01) | -2.9% (-3.4 to -2.4) | <0.001 | 0.79 (0.58 to 0.91) | -2.9% (-3.4 to -2.4) | <0.001 |
| Jordan | 0.04 (0.02 to 0.06) | -4.7% (-5.4 to -3.9) | <0.001 | 2.94 (1.91 to 4.34) | -4.7% (-5.4 to -3.9) | <0.001 |
| Kazakhstan | 0.04 (0.03 to 0.06) | -5.8% (-7.0 to -4.6) | <0.001 | 3.18 (2.09 to 4.57) | -5.8% (-7.0 to -4.6) | <0.001 |
| Kuwait | 0.06 (0.04 to 0.09) | -6.5% (-7.8 to -5.2) | <0.001 | 4.67 (3.19 to 6.66) | -6.5% (-7.8 to -5.2) | <0.001 |
| Kyrgyzstan | 0.12 (0.09 to 0.16) | -4.5% (-6.1 to -3.0) | <0.001 | 9.70 (7.04 to 12.48) | -4.5% (-6.1 to -3.0) | <0.001 |
| Lao People's Democratic Republic | 0.96 (0.61 to 1.41) | -2.2% (-2.5 to -1.9) | <0.001 | 75.57 (47.71 to 110.51) | -2.2% (-2.5 to -1.9) | <0.001 |
| Lebanon | 0.07 (0.03 to 0.14) | -5.7% (-5.9 to -5.5) | <0.001 | 5.71 (2.52 to 11.00) | -5.7% (-5.9 to -5.5) | <0.001 |
| Malaysia | 0.14 (0.09 to 0.21) | -6.0% (-7.1 to -5.0) | <0.001 | 10.68 (6.76 to 15.88) | -6.1% (-7.1 to -5.0) | <0.001 |
| Maldives | 0.21 (0.07 to 0.34) | -6.9% (-8.0 to -5.8) | <0.001 | 16.13 (5.74 to 26.84) | -6.9% (-8.0 to -5.8) | <0.001 |
| Mongolia | 0.11 (0.06 to 0.18) | -3.0% (-4.0 to -1.9) | <0.001 | 8.72 (4.86 to 13.87) | -3.0% (-4.0 to -2.0) | <0.001 |
| Myanmar | 0.23 (0.14 to 0.37) | -4.7% (-4.8 to -4.5) | <0.001 | 18.33 (10.95 to 29.52) | -4.7% (-4.8 to -4.6) | <0.001 |
| Nepal | 0.35 (0.21 to 0.52) | -3.4% (-3.7 to -3.2) | <0.001 | 27.16 (16.26 to 40.53) | -3.4% (-3.7 to -3.2) | <0.001 |
| Oman | 0.03 (0.02 to 0.03) | -5.2% (-5.8 to -4.6) | <0.001 | 1.99 (1.41 to 2.71) | -5.2% (-5.8 to -4.6) | <0.001 |
| Pakistan | 1.43 (0.94 to 1.96) | 0.8% (0.4 to 1.2) | <0.001 | 112.13 (73.27 to 154.04) | 0.8% (0.4 to 1.2) | <0.001 |
| Palestine | 0.02 (0.01 to 0.03) | -6.3% (-7.7 to -4.9) | <0.001 | 1.72 (1.14 to 2.73) | -6.3% (-7.7 to -4.9) | <0.001 |
| Philippines | 1.09 (0.85 to 1.26) | 2.0% (1.3 to 2.7) | <0.001 | 85.38 (67.03 to 98.96) | 2.0% (1.3 to 2.7) | <0.001 |
| Qatar | 0.04 (0.02 to 0.07) | -7.8% (-9.3 to -6.3) | <0.001 | 3.34 (1.80 to 5.56) | -7.8% (-9.4 to -6.3) | <0.001 |
| Republic of Korea | 0.01 (0.01 to 0.01) | -7.6% (-8.3 to -6.9) | <0.001 | 0.79 (0.51 to 1.13) | -7.6% (-8.2 to -6.9) | <0.001 |
| Saudi Arabia | 0.04 (0.02 to 0.06) | -7.1% (-7.4 to -6.8) | <0.001 | 3.00 (1.65 to 4.92) | -7.2% (-7.4 to -6.9) | <0.001 |
| Singapore | 0.02 (0.01 to 0.03) | -7.8% (-9.2 to -6.4) | <0.001 | 1.47 (1.02 to 2.19) | -7.8% (-9.2 to -6.4) | <0.001 |
| Sri Lanka | 0.08 (0.04 to 0.14) | -4.2% (-8.2 to 0.0) | 0.048 | 6.59 (3.41 to 10.54) | -4.4% (-7.1 to -1.6) | 0.002 |
| Syrian Arab Republic | 0.49 (0.31 to 0.71) | -6.0% (-7.0 to -5.1) | <0.001 | 38.43 (24.06 to 55.80) | -6.1% (-7.0 to -5.1) | <0.001 |
| Tajikistan | 0.14 (0.09 to 0.20) | -3.6% (-4.3 to -3.0) | <0.001 | 10.77 (6.73 to 15.85) | -3.6% (-4.3 to -3.0) | <0.001 |
| Thailand | 0.14 (0.10 to 0.21) | -4.5% (-5.3 to -3.7) | <0.001 | 11.33 (7.56 to 16.11) | -4.5% (-5.3 to -3.7) | <0.001 |
| Timor-Leste | 0.68 (0.40 to 1.04) | -0.7% (-1.1 to -0.3) | 0.001 | 53.40 (31.48 to 81.20) | -0.7% (-1.1 to -0.3) | <0.001 |
| Turkey | 0.02 (0.01 to 0.03) | -4.0% (-4.9 to -3.0) | <0.001 | 1.66 (1.09 to 2.45) | -4.0% (-4.9 to -3.0) | <0.001 |
| Turkmenistan | 0.18 (0.12 to 0.26) | -3.5% (-4.7 to -2.4) | <0.001 | 13.95 (9.44 to 20.50) | -3.6% (-4.7 to -2.4) | <0.001 |
| United Arab Emirates | 0.17 (0.09 to 0.29) | -5.6% (-5.9 to -5.3) | <0.001 | 13.52 (7.35 to 22.25) | -5.6% (-5.9 to -5.4) | <0.001 |
| Uzbekistan | 0.25 (0.18 to 0.33) | -3.2% (-4.2 to -2.3) | <0.001 | 19.46 (14.14 to 25.72) | -3.2% (-4.2 to -2.3) | <0.001 |
| Viet Nam | 0.06 (0.04 to 0.09) | -3.8% (-4.0 to -3.7) | <0.001 | 4.43 (2.78 to 7.10) | -3.8% (-4.0 to -3.7) | <0.001 |
| Yemen | 0.55 (0.32 to 0.89) | -2.8% (-3.2 to -2.4) | <0.001 | 43.78 (25.18 to 70.61) | -2.8% (-3.2 to -2.5) | <0.001 |
|  | **15-49 years** |  |  |  |  |  |
|  | Death | | | Years of life lost (YLLs) | | |
| Location Name | rate per 100 000 population, 2019 | Average annual percent change (95% CI), 1990-2019 | P-value | rates per 100 000 population, 2019 | Average annual percent change (95% CI), 1990-2019 | P-value |
| Global | 1.72 (1.43 to 1.98) | -2.5% (-2.6 to -2.3) | <0.001 | 91.99 (77.51 to 105.07) | -2.5% (-2.7 to -2.3) | <0.001 |
| Asia | 2.49 (2.00 to 2.90) | -2.3% (-2.5 to -2.1) | <0.001 | 132.59 (108.00 to 153.44) | -2.3% (-2.5 to -2.1) | <0.001 |
| Central Asia | 2.47 (2.04 to 3.00) | -2.8% (-3.2 to -2.4) | <0.001 | 128.12 (106.51 to 154.82) | -2.9% (-3.6 to -2.3) | <0.001 |
| East Asia | 0.65 (0.53 to 0.76) | -5.9% (-6.3 to -5.4) | <0.001 | 31.36 (25.69 to 37.00) | -6.1% (-6.6 to -5.6) | <0.001 |
| High-income Asia Pacific | 0.08 (0.07 to 0.08) | -4.6% (-4.7 to -4.4) | <0.001 | 3.74 (3.42 to 4.04) | -4.6% (-4.8 to -4.5) | <0.001 |
| South Asia | 4.96 (3.82 to 5.93) | -2.0% (-2.2 to -1.7) | <0.001 | 265.79 (208.44 to 315.37) | -2.1% (-2.3 to -1.8) | <0.001 |
| Southeast Asia | 0.88 (0.74 to 1.02) | -2.6% (-2.9 to -2.4) | <0.001 | 48.65 (40.75 to 56.34) | -2.6% (-2.9 to -2.4) | <0.001 |
| Afghanistan | 2.74 (1.78 to 4.14) | -2.6% (-2.8 to -2.5) | <0.001 | 144.48 (94.34 to 219.60) | -2.8% (-3.0 to -2.6) | <0.001 |
| Armenia | 1.06 (0.73 to 1.50) | -3.9% (-4.9 to -2.8) | <0.001 | 53.10 (36.78 to 74.36) | -3.9% (-4.6 to -3.3) | <0.001 |
| Azerbaijan | 1.13 (0.78 to 1.56) | -3.0% (-3.6 to -2.4) | <0.001 | 60.07 (41.54 to 82.09) | -3.2% (-4.0 to -2.4) | <0.001 |
| Bahrain | 0.27 (0.19 to 0.36) | -2.3% (-2.7 to -1.8) | <0.001 | 13.57 (9.84 to 18.27) | -2.7% (-3.2 to -2.1) | <0.001 |
| Bangladesh | 1.39 (1.02 to 1.82) | -2.7% (-3.3 to -2.1) | <0.001 | 78.84 (57.83 to 103.23) | -2.9% (-3.5 to -2.2) | <0.001 |
| Bhutan | 3.87 (1.90 to 6.98) | -3.0% (-3.3 to -2.7) | <0.001 | 207.60 (103.15 to 375.48) | -3.1% (-3.4 to -2.8) | <0.001 |
| Brunei Darussalam | 0.55 (0.44 to 0.68) | -2.4% (-2.6 to -2.2) | <0.001 | 27.84 (22.55 to 34.49) | -2.6% (-2.9 to -2.3) | <0.001 |
| Cambodia | 2.03 (1.22 to 3.02) | -3.6% (-3.8 to -3.4) | <0.001 | 111.39 (67.37 to 164.20) | -3.7% (-3.9 to -3.5) | <0.001 |
| China | 0.62 (0.50 to 0.74) | -6.0% (-6.5 to -5.6) | <0.001 | 30.17 (24.37 to 35.99) | -6.2% (-6.8 to -5.7) | <0.001 |
| Cyprus | 0.11 (0.09 to 0.14) | -3.4% (-3.7 to -3.0) | <0.001 | 5.17 (4.15 to 6.37) | -3.4% (-3.8 to -3.0) | <0.001 |
| Democratic People's Republic of Korea | 2.30 (1.24 to 3.70) | -1.2% (-1.3 to -1.1) | <0.001 | 113.36 (61.79 to 182.22) | -1.3% (-1.4 to -1.2) | <0.001 |
| Georgia | 1.93 (1.49 to 2.52) | -0.9% (-1.7 to -0.1) | 0.022 | 100.14 (76.67 to 130.63) | -1.2% (-2.3 to 0.0) | 0.048 |
| India | 4.91 (5.99 to 3.81) | -2.2% (-2.5 to -1.9) | <0.001 | 260.62 (206.45 to 314.98) | -2.3% (-2.7 to -1.9) | <0.001 |
| Indonesia | 0.32 (0.26 to 0.40) | -4.6% (-4.7 to -4.4) | <0.001 | 17.36 (14.34 to 21.54) | -4.6% (-4.7 to -4.5) | <0.001 |
| Iran (Islamic Republic of) | 0.43 (0.37 to 0.51) | -3.2% (-3.6 to -2.8) | <0.001 | 22.60 (19.63 to 26.61) | -3.5% (-3.9 to -3.1) | <0.001 |
| Iraq | 0.48 (0.33 to 0.68) | -4.9% (-5.3 to -4.5) | <0.001 | 24.50 (34.64 to 17.20) | -5.0% (-5.4 to -4.6) | <0.001 |
| Israel | 0.27 (0.20 to 0.35) | -2.5% (-3.5 to -1.5) | <0.001 | 13.40 (10.22 to 17.49) | -2.6% (-3.6 to -1.6) | <0.001 |
| Japan | 0.08 (0.07 to 0.08) | -4.3% (-4.5 to -4.0) | <0.001 | 3.74 (4.08 to 3.40) | -4.2% (-4.5 to -4.0) | <0.001 |
| Jordan | 0.13 (0.10 to 0.17) | -4.3% (-4.8 to -3.8) | <0.001 | 7.12 (5.53 to 8.94) | -4.4% (-5.0 to -3.9) | <0.001 |
| Kazakhstan | 1.05 (0.69 to 1.50) | -5.5% (-5.8 to -5.1) | <0.001 | 52.28 (34.57 to 74.27) | -5.7% (-6.0 to -5.4) | <0.001 |
| Kuwait | 0.14 (0.09 to 0.20） | -5.0% (-6.8 to -3.2) | <0.001 | 7.31 (4.99 to 10.27) | -5.3% (-7.0 to -3.5) | <0.001 |
| Kyrgyzstan | 2.31 (1.62 to 3.12) | -4.2% (-4.7 to -3.7) | <0.001 | 118.19 (83.33 to 157.81) | -4.4% (-5.1 to -3.7) | <0.001 |
| Lao People's Democratic Republic | 2.91 (1.93 to 4.08) | -2.2% (-2.4 to -2.0) | <0.001 | 164.12 (108.76 to 229.35) | -2.2% (-2.4 to -2.0) | <0.001 |
| Lebanon | 0.35 (0.16 to 0.55) | -4.2% (-4.3 to -4.1) | <0.001 | 18.65 (8.33 to 29.17) | -4.3% (-4.5 to -4.2) | <0.001 |
| Malaysia | 0.44 (0.32 to 0.60) | -5.5% (-5.7 to -5.2) | <0.001 | 24.29 (17.26 to 32.64) | -5.6% (-5.8 to -5.3) | <0.001 |
| Maldives | 0.42 (0.29 to 0.54) | -6.5% (-6.7 to -6.3) | <0.001 | 22.34 (15.16 to 28.93) | -6.7% (-6.9 to -6.4) | <0.001 |
| Mongolia | 2.27 (1.61 to 3.08) | -1.7% (-2.1 to -1.4) | <0.001 | 111.99 (79.00 to 151.09) | -1.8% (-2.1 to -1.6) | <0.001 |
| Myanmar | 1.40 (0.91 to 2.03) | -3.4% (-3.5 to -3.2) | <0.001 | 75.49 (49.14 to 108.51) | -3.5% (-3.7 to -3.3) | <0.001 |
| Nepal | 4.18 (2.62 to 6.05) | -3.2% (-3.3 to -3.0) | <0.001 | 222.28 (141.92 to 319.05) | -3.2% (-3.4 to -3.0) | <0.001 |
| Oman | 0.05 (0.04 to 0.09) | -5.5% (-6.1 to -4.9) | <0.001 | 2.96 (1.91 to 4.89) | -5.5% (-6.2 to -4.8) | <0.001 |
| Pakistan | 8.15 (5.54 to 10.83) | -0.9% (-1.0 to -0.7) | <0.001 | 451.61 (312.24 to 595.36) | -0.8% (-1.0 to -0.7) | <0.001 |
| Palestine | 0.18 (0.14 to 0.22) | -4.2% (-4.5 to -3.9) | <0.001 | 9.28 (7.28 to 11.58) | -4.3% (-4.7 to -3.9) | <0.001 |
| Philippines | 2.46 (1.85 to 3.03) | 2.2% (1.6 to 2.8) | <0.001 | 140.92 (105.87 to 172.88) | 2.2% (1.6 to 2.8) | <0.001 |
| Qatar | 0.15 (0.10 to 0.21) | -5.4% (-5.8 to -5.0) | <0.001 | 7.74 (5.29 to 11.05) | -5.5% (-5.9 to -5.1) | <0.001 |
| Republic of Korea | 0.06 (0.05 to 0.08) | -5.0% (-5.4 to -4.6) | <0.001 | 3.17 (2.59 to 3.86） | -5.3% (-5.7 to -4.8) | <0.001 |
| Saudi Arabia | 0.56 (0.35 to 0.85) | -3.6% (-4.1 to -3.0) | <0.001 | 28.60 (17.73 to 42.81) | -3.8% (-4.3 to -3.2) | <0.001 |
| Singapore | 0.13 (0.09 to 0.18) | -6.8% (-7.1 to -6.5) | <0.001 | 6.53 (4.76 to 8.98） | -7.0% (-7.3 to -6.6) | <0.001 |
| Sri Lanka | 0.37 (0.25 to 0.53) | -4.9% (-5.6 to -4.2) | <0.001 | 19.90 (13.47 to 28.62) | -5.0% (-5.8 to -4.1) | <0.001 |
| Syrian Arab Republic | 0.61 (0.41 to 0.91) | -6.7% (-7.4 to -6.1) | <0.001 | 32.77 (21.91 to 47.96) | -6.9% (-7.5 to -6.4) | <0.001 |
| Tajikistan | 2.32 (1.80 to 2.99) | -3.5% (-3.9 to -3.1) | <0.001 | 122.62 (95.67 to 157.50) | -3.7% (-4.1 to -3.3) | <0.001 |
| Thailand | 0.25 (0.17 to 0.35) | -7.9% (-8.8 to -7.0) | <0.001 | 12.89 (8.88 to 18.13) | -7.9% (-8.9 to -7.0) | <0.001 |
| Timor-Leste | 1.91 (0.65 to 2.84) | -1.6% (-2.3 to -0.8) | <0.001 | 110.05 (39.06 to 163.00) | -1.4% (-2.2 to -0.6) | 0.001 |
| Turkey | 0.14 (0.11 to 0.18) | -3.9% (-4.4 to -3.3) | <0.001 | 7.04 (5.31 to 9.07) | -4.0% (-4.5 to -3.5) | <0.001 |
| Turkmenistan | 2.40 (1.67 to 3.39) | -2.4% (-3.4 to -1.5) | <0.001 | 129.91 (90.80 to 181.35) | -2.6% (-3.6 to -1.6) | <0.001 |
| United Arab Emirates | 0.82 (0.44 to 1.43) | -1.7% (-1.9 to -1.4) | <0.001 | 39.65 (21.64 to 69.36) | -1.8% (-2.1 to -1.5) | <0.001 |
| Uzbekistan | 3.87 (2.81 to 5.16) | -2.3% (-2.9 to -1.7) | <0.001 | 201.39 (147.48 to 265.92) | -2.6% (-3.2 to -2.0) | <0.001 |
| Viet Nam | 0.69 (0.46 to 0.96) | -3.0% (-3.1 to -2.8) | <0.001 | 35.24 (23.65 to 48.28) | -3.2% (-3.3 to -3.1) | <0.001 |
| Yemen | 1.37 (0.73 to 2.34) | -2.4% (-2.7 to -2.2) | <0.001 | 72.21 (35.58 to 121.63) | -2.4% (-2.7 to -2.1) | <0.001 |
|  | **50-69 years** |  |  |  |  |  |
|  | Death | | | Years of life lost (YLLs) | | |
| Location Name | rate per 100 000  population, 2019 | Average annual  percent change  (95% CI), 1990-2019 | P-value | rates per 100 000  population, 2019 | Average annual percent change (95% CI), 1990-2019 | P-value |
| Global | 6.99 (5.64 to 8.04) | -3.5% (-3.7 to -3.3) | <0.001 | 205.60 (164.65 to 237.30) | -3.5% (-3.7 to -3.4) | <0.001 |
| Asia | 9.70 (7.46 to 11.43) | -3.6% (-3.8 to -3.3) | <0.001 | 286.31 (217.71 to 338.14) | -3.6% (-3.8 to -3.3) | <0.001 |
| Central Asia | 10.09 (8.36 to 11.93) | -2.0% (-2.2 to -1.8) | <0.001 | 311.75 (257.15 to 370.42) | -2.1% (-2.3 to -1.9) | <0.001 |
| East Asia | 5.56 (4.53 to 6.56) | -5.9% (-6.2 to -5.6) | <0.001 | 158.12 (128.71 to 186.83) | -6.0% (-6.3 to -5.7) | <0.001 |
| High-income Asia Pacific | 0.61 (0.57 to 0.66) | -4.9% (-5.1 to -4.6) | <0.001 | 16.72 (15.52 to 18.17) | -5.0% (-5.4 to -4.7) | <0.001 |
| South Asia | 22.62 (16.07 to 28.12) | -2.2% (-2.6 to -1.7) | <0.001 | 675.01 (475.64 to 843.24) | -2.2% (-2.6 to -1.8) | <0.001 |
| Southeast Asia | 1.72 (1.46 to 2.02) | -4.0% (-4.2 to -3.8) | <0.001 | 52.12 (44.16 to 61.23) | -4.0% (-4.3 to -3.8) | <0.001 |
| Afghanistan | 12.70 (6.90 to 19.90) | -2.2% (-2.3 to -2.1) | <0.001 | 403.59 (218.10 to 628.43) | -2.0% (-2.1 to -2.0) | <0.001 |
| Armenia | 6.76 (4.75 to 9.17) | -4.2% (-4.7 to -3.6) | <0.001 | 195.60 (136.89 to 266.44) | -4.3% (-5.2 to -3.5） | <0.001 |
| Azerbaijan | 3.78 (2.74 to 5.03) | -2.7% (-3.1 to -2.3) | <0.001 | 116.11 (83.53 to 155.15) | -2.8% (-3.1 to -2.5) | <0.001 |
| Bahrain | 1.28 (0.96 to 1.70) | -4.3% (-4.7 to -3.8) | <0.001 | 37.77 (28.21 to 49.75) | -4.3% (-4.7 to -3.9) | <0.001 |
| Bangladesh | 3.60 (2.43 to 4.97) | -2.1% (-2.7 to -1.4) | <0.001 | 109.90 (74.16 to 152.69) | -2.1% (-2.7 to -1.4) | <0.001 |
| Bhutan | 23.49 (10.39 to 52.40) | -2.8% (-2.9 to -2.7) | <0.001 | 688.92 (300.62 to 1519.05) | -2.9% (-3.0 to -2.8) | <0.001 |
| Brunei Darussalam | 4.10 (3.23 to 5.11) | -2.5% (-2.7 to -2.4) | <0.001 | 120.82 (94.93 to 150.58) | -2.6% (-2.8 to -2.4) | <0.001 |
| Cambodia | 5.12 (3.00 to 7.43) | -3.5% (-3.6 to -3.4) | <0.001 | 157.69 (91.83 to 230.02) | -3.6% (-3.6 to -3.5) | <0.001 |
| China | 5.49 (4.41 to 6.54) | -6.0% (-6.3 to -5.7) | <0.001 | 155.56 (124.92 to 186.14) | -6.1% (-6.4 to -5.8) | <0.001 |
| Cyprus | 2.81 (2.24 to 3.51) | -4.4% (-4.7 to -4.2) | <0.001 | 75.96 (61.25 to 96.15) | -4.4% (-4.7 to -4.2) | <0.001 |
| Democratic People's Republic of Korea | 16.18 (9.62 to 24.02) | -1.4% (-1.5 to -1.2) | <0.001 | 483.20 (288.49 to 727.75) | -1.3% (-1.5 to -1.2) | <0.001 |
| Georgia | 10.07 (7.09 to 13.46) | 0.6% (0.1 to 1.0) | 0.015 | 287.94 (201.86 to 384.73) | 0.3% (-0.1 to 0.8) | 0.167 |
| India | 23.86 (16.44 to 30.09) | -2.2% (-2.6 to -1.7) | <0.001 | 710.54 (482.27 to 904.60) | -2.2% (-2.6 to -1.7) | <0.001 |
| Indonesia | 0.81 (0.65 to 0.97) | -4.7% (-5.2 to -4.2) | <0.001 | 23.87 (19.23 to 28.86) | -4.8% (-5.3 to -4.3) | <0.001 |
| Iran (Islamic Republic of) | 2.55 (2.20 to 2.97) | -3.4% (-3.7 to -3.1) | <0.001 | 74.10 (63.91 to 85.92) | -3.4% (-3.9 to -2.9) | <0.001 |
| Iraq | 3.09 (2.20 to 4.07) | -4.6% (-4.8 to -4.3) | <0.001 | 93.32 (65.93 to 124.06) | -4.6% (-4.9 to -4.3) | <0.001 |
| Israel | 2.61 (1.91 to 3.42) | -2.8% (-3.7 to -1.9) | <0.001 | 73.32 (53.35 to 96.15) | -2.8% (-3.7 to -1.9) | <0.001 |
| Japan | 0.67 (0.62 to 0.72) | -4.6% (-4.8 to -4.4) | <0.001 | 18.12 (16.85 to 19.57) | -4.8% (-5.0 to -4.6) | <0.001 |
| Jordan | 0.85 (0.64 to 1.09) | -4.8% (-5.1 to -4.6) | <0.001 | 25.37 (19.21 to 32.61) | -4.8% (-5.1 to -4.6) | <0.001 |
| Kazakhstan | 6.53 (4.67 to 8.90) | -4.2% (-4.7 to -3.7) | <0.001 | 197.01 (140.13 to 269.89) | -4.3% (-4.9 to -3.8) | <0.001 |
| Kuwait | 0.51 (0.34 to 0.73) | -6.0% (-7.0 to -4.9) | <0.001 | 15.40 (10.38 to 22.30) | -6.0% (-7.0 to -5.0) | <0.001 |
| Kyrgyzstan | 9.35 (6.88 to 12.54) | -3.1% (-3.6 to -2.5) | <0.001 | 294.76 (216.00 to 396.39) | -3.1% (-3.7 to -2.5) | <0.001 |
| Lao People's Democratic Republic | 5.84 (3.89 to 8.29) | -3.1% (-3.2 to -3.0) | <0.001 | 179.84 (119.15 to 256.43) | -3.1% (-3.2 to -3.1) | <0.001 |
| Lebanon | 1.50 (0.67 to 2.42) | -3.8% (-3.9 to -3.6) | <0.001 | 43.46 (19.48 to 70.59) | -3.8% (-3.9 to -3.7) | <0.001 |
| Malaysia | 1.65 (1.17 to 2.26) | -5.7% (-5.9 to -5.5) | <0.001 | 48.82 (34.46 to 66.81) | -5.7% (-5.9 to -5.5) | <0.001 |
| Maldives | 1.80 (1.39 to 2.31) | -5.8% (-6.1 to -5.6) | <0.001 | 54.36 (41.91 to 69.58) | -5.9% (-6.1 to -5.7) | <0.001 |
| Mongolia | 10.97 (7.97 to 14.82) | -3.8% (-4.0 to -3.6) | <0.001 | 338.25 (245.33 to 459.12) | -3.7% (-3.9 to -3.5) | <0.001 |
| Myanmar | 3.63 (2.56 to 5.01) | -3.5% (-3.6 to -3.4) | <0.001 | 110.46 (77.69 to 153.34) | -3.5% (-3.6 to -3.4) | <0.001 |
| Nepal | 26.09 (15.43 to 40.98) | -2.4% (-2.6 to -2.3) | <0.001 | 765.33 (451.26 to 1190.39) | -2.5% (-2.7 to -2.4) | <0.001 |
| Oman | 0.73 (0.55 to 1.01) | -5.6% (-6.2 to -4.9) | <0.001 | 20.77 (15.45 to 29.16) | -5.6% (-6.1 to -5.0) | <0.001 |
| Pakistan | 30.38 (21.24 to 41.23) | -1.6% (-1.7 to -1.5) | <0.001 | 920.98 (639.21 to 1263.83) | -1.6% (-1.7 to -1.5) | <0.001 |
| Palestine | 1.13 (0.88 to 1.43) | -4.6% (-4.8 to -4.3) | <0.001 | 33.55 (26.24 to 42.14) | -4.5% (-4.7 to -4.3) | <0.001 |
| Philippines | 2.93 (2.31 to 3.59) | 0.4% (-0.3 to 1.0) | 0.265 | 91.04 (71.41 to 111.88) | 0.4% (-0.2 to 1.0) | 0.229 |
| Qatar | 0.69 (0.46 to 0.99) | -6.0% (-6.6 to -5.5) | <0.001 | 21.84 (14.52 to 31.80) | -6.1% (-6.6 to -5.6) | <0.001 |
| Republic of Korea | 0.45 (0.36 to 0.56) | -5.0% (-5.3 to -4.8) | <0.001 | 12.98 (10.35 to 16.11) | -5.2% (-5.4 to -5.0) | <0.001 |
| Saudi Arabia | 1.86 (1.27 to 2.49) | -4.2% (-4.6 to -3.9) | <0.001 | 58.26 (39.46 to 78.48) | -4.2% (-4.7 to -3.6) | <0.001 |
| Singapore | 0.67 (0.46 to 0.92) | -7.3% (-7.7 to -6.8) | <0.001 | 19.59 (13.56 to 27.33) | -7.4% (-7.8 to -6.9) | <0.001 |
| Sri Lanka | 1.33 (0.89 to 1.88) | -4.7% (-5.0 to -4.3) | <0.001 | 38.94 (25.91 to 54.64) | -4.8% (-5.3 to -4.3) | <0.001 |
| Syrian Arab Republic | 3.22 (2.11 to 4.63) | -6.6% (-7.1 to -6.0) | <0.001 | 96.16 (62.61 to 139.79) | -6.6% (-7.2 to -6.0) | <0.001 |
| Tajikistan | 8.60 (6.57 to 11.16) | -3.1% (-3.6 to -2.7) | <0.001 | 266.66 (203.29 to 348.05) | -3.2% (-3.6 to -2.8) | <0.001 |
| Thailand | 0.86 (0.60 to 1.21) | -7.2% (-8.0 to -6.4) | <0.001 | 25.15 (17.44 to 35.37) | -7.3% (-8.1 to -6.5) | <0.001 |
| Timor-Leste | 5.89 (3.60 to 9.13) | -1.6% (-1.9 to -1.3) | <0.001 | 172.62 (105.77 to 269.08) | -1.8% (-2.1 to -1.5) | <0.001 |
| Turkey | 1.18 (0.89 to 1.53) | -4.6% (-5.1 to -4.1) | <0.001 | 34.22 (25.57 to 44.20) | -4.6% (-4.9 to -4.3) | <0.001 |
| Turkmenistan | 4.58 (3.01 to 6.71) | -3.2% (-3.9 to -2.5) | <0.001 | 145.80 (95.44 to 213.96) | -3.2% (-3.9 to -2.5) | <0.001 |
| United Arab Emirates | 7.06 (3.86 to 11.48) | -3.6% (-3.8 to -3.3) | <0.001 | 217.16 (118.90 to 353.40) | -3.6% (-3.8 to -3.3) | <0.001 |
| Uzbekistan | 17.07 (12.26 to 22.87) | -0.4% (-1.0 to 0.1) | 0.124 | 535.80 (383.45 to 721.78) | -0.5% (-1.1 to 0.0) | 0.061 |
| Viet Nam | 2.33 (1.54 to 3.19) | -4.1% (-4.2 to -4.0) | <0.001 | 70.61 (46.73 to 96.84) | -4.0% (-4.1 to -3.9) | <0.001 |
| Yemen | 8.66 (5.31 to 13.75) | -2.3% (-2.5 to -2.1) | <0.001 | 257.61 (158.03 to 410.55) | -2.3% (-2.5 to -2.1) | <0.001 |
|  | **70+ years** |  |  |  |  |  |
|  | Death | | | Years of life lost (YLLs) | | |
| Location Name | rate per 100 000 population, 2019 | Average annual percent change (95% CI), 1990-2019 | P-value | rates per 100 000 population, 2019 | Average annual percent change (95% CI), 1990-2019 | P-value |
| Global | 29.24 (25.15 to 32.36) | -2.4% (-2.5 to -2.2) | <0.001 | 378.66 (324.36 to 419.17) | -2.7% (-2.8 to -2.6) | <0.001 |
| Asia | 40.88 (33.85 to 45.98) | -3.0% (-3.2 to -2.8) | <0.001 | 545.24 (452.30 to 614.12) | -3.3% (-3.5 to -3.1) | <0.001 |
| Central Asia | 19.15 (16.59 to 21.69） | 0.5% (-0.1 to 1.1) | 0.106 | 253.16 (222.11 to 282.78) | 0.2% (-0.2 to 0.7) | 0.330 |
| East Asia | 41.30 (34.59 to 47.38) | -4.1% (-4.5 to -3.7) | <0.001 | 538.62 (450.51 to 620.88) | -4.5% (-4.8 to -4.2) | <0.001 |
| High-income Asia Pacific | 14.26 (10.49 to 16.62) | -1.1% (-1.3 to -0.9) | <0.001 | 130.12 (99.79 to 148.38) | -2.2% (-2.4 to -2.0) | <0.001 |
| South Asia | 71.41 (55.72 to 86.43) | -1.8% (-2.7 to -1.0) | <0.001 | 1003.55 (781.51 to 1218.16) | -2.1% (-2.8 to -1.3） | <0.001 |
| Southeast Asia | 7.70 (6.10 to 8.89) | -3.5% (-3.7 to -3.3) | <0.001 | 99.14 (80.65 to 113.89) | -3.7% (-3.9 to -3.5) | <0.001 |
| Afghanistan | 33.78 (16.18 to 63.86) | -1.5% (-1.5 to -1.4) | <0.001 | 496.48 (237.61 to 938.10) | -1.6% (-1.7 to -1.5) | <0.001 |
| Armenia | 21.64 (17.44 to 26.70) | 0.0 (-0.5 to 0.6) | 0.867 | 278.85 (226.88 to 341.57) | -0.3% (-0.9 to 0.3) | 0.342 |
| Azerbaijan | 11.61 (7.70 to 15.77) | -1.7% (-2.2 to -1.1) | <0.001 | 154.42 (105.06 to 209.06) | -1.4% (-1.9 to -1.0) | <0.001 |
| Bahrain | 9.93 (7.56 to 12.80) | -2.1% (-2.9 to -1.2) | <0.001 | 139.40 (106.42 to 182.43) | -2.4% (-3.2 to -1.5) | <0.001 |
| Bangladesh | 18.06 (11.75 to 24.35) | -1.2% (-1.9 to -0.4) | 0.002 | 217.09 (144.28 to 293.47) | -1.4% (-2.2 to -0.7) | <0.001 |
| Bhutan | 99.56 (46.14 to 200.08) | -1.4% (-1.5 to -1.4) | <0.001 | 1353.74 (621.69 to 2717.07) | -1.7% (-1.8 to -1.7) | <0.001 |
| Brunei Darussalam | 21.02 (17.76 to 24.91) | -1.6% (-1.8 to -1.4) | <0.001 | 299.96 (250.19 to 357.95) | -1.8% (-2.1 to -1.4) | <0.001 |
| Cambodia | 11.50 (7.81 to 14.80) | -2.7% (-2.9 to -2.6) | <0.001 | 167.17 (111.23 to 219.15) | -2.9% (-3.0 to -2.8) | <0.001 |
| China | 41.65 (34.46 to 48.09) | -4.1% (-4.6 to -3.7) | <0.001 | 542.64 (447.83 to 628.75) | -4.5% (-4.8 to -4.3) | <0.001 |
| Cyprus | 31.22 (25.97 to 38.60) | -2.9% (-3.4 to -2.4) | <0.001 | 381.72 (315.40 to 472.42) | -3.4% (-3.8 to -2.9) | <0.001 |
| Democratic People's Republic of Korea | 62.86 (36.40 to 97.36） | -1.5% (-1.6 to -1.4) | <0.001 | 859.38 (498.18 to 1323.56) | -1.7% (-1.8 to -1.6) | <0.001 |
| Georgia | 64.08 (49.79 to 80.07) | 5.1% (3.0 to 7.2) | <0.001 | 738.05 (578.12 to 910.50) | 4.6% (2.9 to 6.3) | <0.001 |
| India | 74.87 (57.26 to 91.28) | -1.8% (-2.9 to -0.7) | 0.001 | 1055.36 (799.99 to 1289.04) | -2.2% (-2.9 to -1.5) | <0.001 |
| Indonesia | 4.92 (4.13 to 5.52) | -3.1% (-3.5 to -2.8) | <0.001 | 66.67 (56.53 to 74.88) | -3.2% (-3.6 to -2.9) | <0.001 |
| Iran (Islamic Republic of) | 13.27 (10.73 to 15.01) | -1.8% (-2.3 to -1.3) | <0.001 | 169.51 (138.76 to 191.35) | -2.3% (-2.8 to -1.8) | <0.001 |
| Iraq | 10.84 (8.50 to 13.08) | -3.6% (-4.1 to -3.1) | <0.001 | 146.67 (114.33 to 176.57) | -3.6% (-4.1 to -3.1) | <0.001 |
| Israel | 19.20 (15.74 to 22.73) | -0.8% (-1.7 to 0.1) | 0.089 | 217.54 (182.32 to 256.30) | -1.4% (-2.3 to -0.6) | 0.001 |
| Japan | 16.32 (11.89 to 19.05) | -0.9% (-1.1 to -0.7) | <0.001 | 146.57 (111.83 to 168.11) | -2.0% (-2.2 to -1.8) | <0.001 |
| Jordan | 3.18 (2.38 to 4.10) | -3.8% (-4.3 to -3.4) | <0.001 | 44.89 (33.50 to 57.83) | -3.9% (-4.3 to -3.5) | <0.001 |
| Kazakhstan | 7.25 (5.78 to 8.81) | -2.4% (-2.7 to -2.1) | <0.001 | 109.84 (87.44 to 134.95) | -2.5% (-2.8 to -2.1) | <0.001 |
| Kuwait | 4.24 (3.09 to 5.67) | -3.9% (-5.3 to -2.6) | <0.001 | 49.50 (36.49 to 65.22) | -4.2% (-5.5 to -2.8) | <0.001 |
| Kyrgyzstan | 5.60 (4.53 to 6.90) | -2.5% (-2.9 to -2.1) | <0.001 | 78.88 (63.62 to 97.57) | -2.7% (-3.1 to -2.3) | <0.001 |
| Lao People's Democratic Republic | 14.52 (10.31 to 19.02) | -2.2% (-2.3 to -2.2) | <0.001 | 208.81 (145.41 to 278.54) | -2.5% (-2.6 to -2.5) | <0.001 |
| Lebanon | 6.65 (2.97 to 10.13) | -3.4% (-3.6 to -3.3) | <0.001 | 87.01 (38.65 to 133.87) | -3.6% (-3.8 to -3.5) | <0.001 |
| Malaysia | 5.13 (3.69 to 6.55) | -4.7% (-5.4 to -4.0) | <0.001 | 74.33 (51.99 to 96.82) | -4.8% (-5.3 to -4.3) | <0.001 |
| Maldives | 10.73 (8.22 to 13.14) | -3.1% (-3.4 to -2.9) | <0.001 | 135.10 (103.92 to 167.27) | -3.7% (-4.0 to -3.4) | <0.001 |
| Mongolia | 32.99 (25.53 to 41.86) | -3.0% (-3.3 to -2.7) | <0.001 | 454.74 (346.11 to 582.97) | -3.2% (-3.4 to -2.9) | <0.001 |
| Myanmar | 15.17 (11.75 to 19.25) | -2.1% (-2.1 to -2.0) | <0.001 | 199.08 (154.40 to 255.65) | -2.4% (-2.5 to -2.4) | <0.001 |
| Nepal | 99.00 (63.56 to 168.45) | -1.2% (-1.3 to -1.2) | <0.001 | 1397.33 (867.04 to 2366.80) | -1.4% (-1.5 to -1.3) | <0.001 |
| Oman | 5.48 (4.16 to 6.80) | -3.6% (-4.0 to -3.3) | <0.001 | 80.24 (61.19 to 100.19) | -3.7% (-4.2 to -3.2) | <0.001 |
| Pakistan | 103.51 (79.23 to 128.92) | -1.3% (-1.4 to -1.2) | <0.001 | 1470.00 (1122.64 to 1840.35) | -1.3% (-1.4 to -1.1) | <0.001 |
| Palestine | 6.32 (4.78 to 7.59) | -3.1% (-3.4 to -2.8) | <0.001 | 83.07 (62.32 to 100.74) | -3.2% (-3.5 to -2.9) | <0.001 |
| Philippines | 6.09 (5.03 to 7.19) | -0.6% (-1.1 to -0.1) | 0.020 | 86.49 (71.35 to 102.34) | -0.6% (-1.0 to -0.1) | 0.014 |
| Qatar | 5.80 (4.34 to 7.57) | -4.5% (-5.2 to -3.8) | <0.001 | 86.31 (63.87 to 114.03) | -4.3% (-5.0 to -3.6) | <0.001 |
| Republic of Korea | 4.35 (3.47 to 5.35) | -2.4% (-2.8 to -2.0) | <0.001 | 50.90 (41.26 to 62.17) | -3.1% (-3.5 to -2.7) | <0.001 |
| Saudi Arabia | 4.83 (3.35 to 6.25) | -3.7% (-4.1 to -3.3) | <0.001 | 70.16 (48.79 to 91.22) | -3.8% (-4.2 to -3.4) | <0.001 |
| Singapore | 2.92 (2.25 to 3.62) | -5.0% (-5.5 to -4.5) | <0.001 | 36.97 (28.85 to 45.56) | -5.4% (-5.6 to -5.3) | <0.001 |
| Sri Lanka | 6.20 (4.20 to 8.16) | -3.8% (-4.3 to -3.2) | <0.001 | 83.04 (56.34 to 111.23) | -4.0% (-4.5 to -3.4) | <0.001 |
| Syrian Arab Republic | 10.18 (7.24 to 13.81) | -5.1% (-5.3 to -4.8) | <0.001 | 149.52 (104.88 to 206.33) | -5.0% (-5.5 to -4.6) | <0.001 |
| Tajikistan | 13.73 (10.30 to 17.37) | -0.8% (-1.5 to -0.2) | 0.008 | 205.97 (153.85 to 264.89) | -0.8% (-1.3 to -0.3) | 0.002 |
| Thailand | 3.66 (2.66 to 4.75) | -7.1% (-7.6 to -6.6) | <0.001 | 46.93 (34.06 to 61.36) | -7.2% (-7.7 to -6.7) | <0.001 |
| Timor-Leste | 14.62 (9.64 to 23.40) | -1.5% (-1.7 to -1.3) | <0.001 | 219.19 (141.82 to 353.75) | -1.3% (-1.5 to -1.2) | <0.001 |
| Turkey | 5.37 (4.15 to 6.64) | -2.7% (-3.1 to -2.4) | <0.001 | 69.67 (53.27 to 86.84) | -2.9% (-3.2 to -2.6) | <0.001 |
| Turkmenistan | 6.70 (5.01 to 8.65) | -3.0% (-3.8 to -2.2) | <0.001 | 91.43 (69.11 to 118.82) | -3.1% (-3.9 to -2.3) | <0.001 |
| United Arab Emirates | 22.76 (13.36 to 36.26) | -3.2% (-3.8 to -2.7) | <0.001 | 365.95 (205.53 to 604.64) | -3.2% (-3.9 to -2.5) | <0.001 |
| Uzbekistan | 15.88 (12.58 to 19.64) | 0.9% (0.3 to 1.4) | 0.001 | 255.67 (202.68 to 315.53) | 1.3% (0.7 to 1.9) | <0.001 |
| Viet Nam | 17.33 (11.04 to 22.19) | -2.9% (-3.0 to -2.8) | <0.001 | 199.80 (130.42 to 257.76) | -3.2% (-3.2 to -3.1) | <0.001 |
| Yemen | 25.38 (16.93 to 38.09) | -1.7% (-1.8 to -1.6) | <0.001 | 371.49 (244.70 to 562.50) | -1.9% (-2.0 to -1.8) | <0.001 |
|  | **Age-standardized** |  |  |  |  |  |
|  | Death | | | Years of life lost (YLLs) | | |
| Location Name | rate per 100 000 population, 2019 | Average annual percent change (95% CI), 1990-2019 | P-value | rates per 100 000 population, 2019 | Average annual percent change (95% CI), 1990-2019 | P-value |
| Global | 3.85 (3.29 to 4.29) | -2.9% (-3.0 to -2.8) | <0.001 | 107.67 (92.66 to 120.94) | -3.0% (-3.2 to -2.9) | <0.001 |
| Asia | 5.51 (4.51 to 6.25) | -3.2% (-3.3 to -3.1) | <0.001 | 151.96 (125.44 to 173.31) | -3.1% (-3.3 to -3.0) | <0.001 |
| Central Asia | 4.16 (3.64 to 4.69) | -1.9% (-2.2 to -1.5) | <0.001 | 131.36 (112.63 to 151.17) | -2.6% (-2.9 to -2.3) | <0.001 |
| East Asia | 4.01 (3.34 to 4.63) | -5.0% (-5.4 to -4.7) | <0.001 | 73.88 (61.34 to 85.97) | -5.7% (-5.9 to -5.4) | <0.001 |
| High-income Asia Pacific | 0.81 (0.64 to 0.93) | -3.0% (-3.1 to -2.9) | <0.001 | 11.20 (9.66 to 12.22) | -3.9% (-4.1 to -3.7) | <0.001 |
| South Asia | 11.18 (8.62 to 13.40) | -2.1% (-2.7 to -1.6) | <0.001 | 316.97 (243.57 to 380.96) | -2.2% (-2.5 to -1.9) | <0.001 |
| Southeast Asia | 1.30 (1.11 to 1.48) | -3.5% (-3.6 to -3.3) | <0.001 | 44.71 (38.81 to 51.01) | -3.2% (-3.4 to -3.0) | <0.001 |
| Afghanistan | 6.15 (3.61 to 9.65) | -2.0% (-2.1 to -2.0) | <0.001 | 186.52 (118.01 to 277.88) | -2.4% (-2.5 to -2.2) | <0.001 |
| Armenia | 2.81 (2.25 to 3.42) | -3.1% (-3.7 to -2.5) | <0.001 | 72.54 (56.28 to 91.13) | -3.9% (-4.5 to -3.3) | <0.001 |
| Azerbaijan | 2.06 (1.58 to 2.69) | -2.3% (-2.6 to -2.0) | <0.001 | 60.48 (45.85 to 78.38) | -2.9% (-3.4 to -2.5) | <0.001 |
| Bahrain | 1.12 (0.87 to 1.41) | -2.7% (-3.1 to -2.3) | <0.001 | 23.64 (18.68 to 30.18) | -3.2% (-3.9 to -2.5) | <0.001 |
| Bangladesh | 2.74 (1.93 to 3.57) | -1.9% (-2.6 to -1.2) | <0.001 | 78.69 (58.54 to 100.30) | -2.2% (-3.3 to -1.1) | <0.001 |
| Bhutan | 12.50 (6.04 to 25.04) | -2.4% (-2.4 to -2.3) | <0.001 | 310.50 (155.48 to 611.28) | -2.8% (-2.9 to -2.7) | <0.001 |
| Brunei Darussalam | 2.65 (2.28 to 3.04) | -1.9% (-2.2 to -1.6) | <0.001 | 54.67 (46.45 to 63.38) | -2.4% (-2.5 to -2.3) | <0.001 |
| Cambodia | 2.71 (1.76 to 3.73) | -3.5% (-3.6 to -3.4) | <0.001 | 98.97 (63.36 to 140.39) | -3.8% (-4.0 to -3.6) | <0.001 |
| China | 4.04 (3.32 to 4.69) | -5.1% (-5.4 to -4.7) | <0.001 | 73.33 (60.04 to 85.94) | -5.8% (-6.0 to -5.5) | <0.001 |
| Cyprus | 2.77 (2.33 to 3.38) | -3.5% (-3.9 to -3.1) | <0.001 | 39.34 (33.43 to 47.09) | -3.9% (-4.2 to -3.5) | <0.001 |
| Democratic People's Republic of Korea | 7.90 (4.81 to 11.54) | -1.5% (-1.7 to -1.4) | <0.001 | 185.64 (114.50 to 277.14) | -1.6% (-1.7 to -1.5) | <0.001 |
| Georgia | 6.03 (4.84 to 7.33) | 1.4% (1.0 to 1.9) | <0.001 | 145.27 (117.27 to 175.60) | 0.1% (-0.4 to 0.5) | 0.805 |
| India | 11.51 (8.63 to 14.03) | -2.3% (-3.0 to -1.6) | <0.001 | 319.42 (244.66 to 390.49) | -2.4% (-2.8 to -2.0) | <0.001 |
| Indonesia | 0.67 (0.57 to 0.76) | -4.0% (-4.2 to -3.7) | <0.001 | 18.79 (16.08 to 21.97) | -4.5% (-4.7 to -4.4) | <0.001 |
| Iran (Islamic Republic of) | 1.48 (1.26 to 1.67) | -3.1% (-3.5 to -2.7) | <0.001 | 36.67 (32.18 to 41.48) | -3.7% (-4.1 to -3.4) | <0.001 |
| Iraq | 1.53 (1.19 to 1.90) | -4.3% (-4.7 to -3.9) | <0.001 | 39.34 (29.98 to 50.88) | -4.8% (-5.0 to -4.6) | <0.001 |
| Israel | 1.63 (1.37 to 1.89) | -1.8% (-2.6 to -1.1) | <0.001 | 31.00 (26.28 to 36.65) | -2.4% (-3.4 to -1.5) | <0.001 |
| Japan | 0.88 (0.68 to 1.00) | -2.8% (-3.2 to -2.5) | <0.001 | 11.94 (10.20 to 13.13) | -3.6% (-4.0 to -3.3) | <0.001 |
| Jordan | 0.45 (0.35 to 0.56) | -4.2% (-4.6 to -3.9) | <0.001 | 11.40 (9.04 to 14.12) | -4.6% (-4.9 to -4.3) | <0.001 |
| Kazakhstan | 1.99 (1.51 to 2.53) | -4.5% (-4.9 to -4.1) | <0.001 | 63.08 (47.14 to 81.40) | -5.0% (-5.4 to -4.6) | <0.001 |
| Kuwait | 0.43 (0.33 to 0.55) | -4.9% (-5.7 to -4.1) | <0.001 | 10.35 (8.10 to 13.27) | -5.4% (-6.4 to -4.5) | <0.001 |
| Kyrgyzstan | 3.05 (2.39 to 3.78) | -3.7% (-4.4 to -2.9) | <0.001 | 113.90 (89.45 to 142.44) | -4.0% (-4.8 to -3.2) | <0.001 |
| Lao People's Democratic Republic | 3.58 (2.51 to 4.72) | -2.6% (-2.8 to -2.5) | <0.001 | 139.54 (95.87 to 189.13) | -2.6% (-2.8 to -2.5) | <0.001 |
| Lebanon | 0.84 (0.39 to 1.28) | -3.9% (-4.0 to -3.8) | <0.001 | 22.74 (10.76 to 34.54) | -4.2% (-4.3 to -4.1) | <0.001 |
| Malaysia | 0.85 (0.62 to 1.08) | -5.4% (-5.8 to -4.9) | <0.001 | 26.37 (19.16 to 34.25) | -5.6% (-5.9 to -5.4) | <0.001 |
| Maldives | 1.21 (0.98 to 1.45) | -5.2% (-5.3 to -5.0) | <0.001 | 32.04 (24.78 to 39.40) | -6.2% (-6.5 to -5.9) | <0.001 |
| Mongolia | 5.42 (4.25 to 6.78) | -3.1% (-3.3 to -2.9) | <0.001 | 139.43 (105.19 to 181.22) | -3.2% (-3.3 to -3.1) | <0.001 |
| Myanmar | 2.38 (1.84 to 3.10) | -3.1% (-3.2 to -3.0) | <0.001 | 75.53 (54.41 to 103.31) | -3.6% (-3.7 to -3.5) | <0.001 |
| Nepal | 13.36 (8.76 to 20.49) | -2.1% (-2.2 to -2.0) | <0.001 | 332.87 (215.86 to 484.76) | -2.6% (-2.7 to -2.5) | <0.001 |
| Oman | 0.60 (0.49 to 0.72) | -4.0% (-4.4 to -3.5) | <0.001 | 11.31 (9.32 to 13.59) | -4.6% (-4.9 to -4.3) | <0.001 |
| Pakistan | 16.90 (12.76 to 21.44) | -1.2% (-1.3 to -1.1) | <0.001 | 504.35 (371.13 to 656.07) | -1.2% (-1.3 to -1.1) | <0.001 |
| Palestine | 0.74 (0.58 to 0.89) | -3.6% (-3.9 to -3.4) | <0.001 | 16.61 (13.36 to 19.88) | -4.2% (-4.4 to -4.1) | <0.001 |
| Philippines | 2.33 (1.82 to 2.79) | 0.9% (0.4 to 1.5) | <0.001 | 107.52 (83.32 to 128.98) | 1.5% (1.0 to 1.9) | <0.001 |
| Qatar | 0.83 (0.64 to 1.05) | -4.4% (-5.1 to -3.7) | <0.001 | 15.97 (12.05 to 20.94) | -5.2% (-5.5 to -4.9) | <0.001 |
| Republic of Korea | 0.38 (0.31 to 0.46) | -3.8% (-4.1 to -3.5) | <0.001 | 6.80 (5.73 to 7.98) | -4.8% (-4.9 to -4.7) | <0.001 |
| Saudi Arabia | 0.91 (0.64 to 1.19) | -4.1% (-4.4 to -3.7) | <0.001 | 27.74 (19.07 to 37.47) | -4.3% (-4.7 to -4.0) | <0.001 |
| Singapore | 0.34 (0.28 to 0.42) | -6.3% (-6.6 to -6.1) | <0.001 | 8.56 (7.01 to 10.53) | -6.9% (-7.2 to -6.7) | <0.001 |
| Sri Lanka | 0.83 (0.58 to 1.11) | -4.4% (-4.8 to -3.9) | <0.001 | 22.39 (15.54 to 30.67) | -4.7% (-5.3 to -4.2) | <0.001 |
| Syrian Arab Republic | 1.66 (1.17 to 2.25) | -6.0% (-6.4 to -5.6) | <0.001 | 52.75 (36.65 to 72.44) | -6.5% (-7.1 to -6.0) | <0.001 |
| Tajikistan | 3.56 (2.87 to 4.43) | -3.0% (-3.2 to -2.7) | <0.001 | 121.46 (96.72 to 153.13) | -3.5% (-3.9 to -3.1) | <0.001 |
| Thailand | 0.50 (0.37 to 0.66) | -7.5% (-8.1 to -6.9) | <0.001 | 15.47 (11.62 to 20.15) | -7.5% (-8.1 to -6.9) | <0.001 |
| Timor-Leste | 3.07 (2.05 to 4.38) | -1.6% (-1.9 to -1.3) | <0.001 | 108.65 (64.19 to 159.84） | -1.7% (-2.2 to -1.2) | <0.001 |
| Turkey | 0.59 (0.46 to 0.73) | -3.6% (-3.9 to -3.4) | <0.001 | 13.62 (10.72 to 16.98) | -4.1% (-4.4 to -3.9) | <0.001 |
| Turkmenistan | 2.35 (1.75 to 3.11) | -3.2% (-4.2 to -2.1) | <0.001 | 95.44 (70.02 to 126.38) | -3.1% (-4.0 to -2.2) | <0.001 |
| United Arab Emirates | 3.45 (2.08 to 5.28) | -3.4% (-3.9 to -3.0) | <0.001 | 83.90 (49.87 to 128.94) | -3.4% (-3.8 to -3.1) | <0.001 |
| Uzbekistan | 5.79 (4.54 to 7.15) | -1.3% (-1.9 to -0.8) | <0.001 | 204.61 (157.56 to 256.65) | -1.9% (-2.4 to -1.4) | <0.001 |
| Viet Nam | 1.80 (1.22 to 2.29) | -3.5% (-3.5 to -3.4) | <0.001 | 41.41 (28.74 to 53.66) | -3.8% (-3.9 to -3.7) | <0.001 |
| Yemen | 4.04 (2.67 to 6.09) | -2.2% (-2.5 to -1.9) | <0.001 | 117.45 (74.12 to 181.96) | -2.6% (-2.8 to -2.4) | <0.001 |

Appendix table 3: Rheumatic heart disease prevalence rates per 100 000 population in 2019 and its average annual percentage change in the period 1990-2019

| Location Name | Age-standardized prevalence rate (per 100 000 population), 2019 | Average annual percent change (95% CI), 1990-2019 | P-value |
| --- | --- | --- | --- |
| Global | 513.68 | 0.45% | <0.001 |
| Asia | 465.55 | 0.14% | <0.001 |
| Central Asia | 623.66 | 0.19% | <0.001 |
| East Asia | 387.68 | -0.45% | <0.001 |
| High-income Asia Pacific | 34.16 | -1.26% | <0.001 |
| South Asia | 645.12 | 0.14% | <0.001 |
| Southeast Asia | 284.89 | 0.12% | <0.001 |
| Afghanistan | 504.92 | 0.07% | <0.001 |
| Armenia | 797.21 | -0.13% | <0.001 |
| Azerbaijan | 734.34 | -0.06% | <0.001 |
| Bahrain | 36.22 | -0.77% | <0.001 |
| Bangladesh | 562.24 | 0.14% | <0.001 |
| Bhutan | 580.88 | 0.01% | 0.148 |
| Brunei Darussalam | 72.66 | -0.89% | <0.001 |
| Cambodia | 429.21 | -0.04% | 0.03 |
| China | 390.24 | -0.47% | <0.001 |
| Cyprus | 32.88 | -1.66% | <0.001 |
| Democratic People's Republic of Korea | 425.60 | 0.03% | <0.001 |
| Georgia | 768.80 | 0.06% | <0.001 |
| India | 638.92 | 0.10% | <0.001 |
| Indonesia | 148.85 | 0.31% | <0.001 |
| Iran (Islamic Republic of) | 507.36 | 0.05% | <0.001 |
| Iraq | 524.30 | 0.05% | <0.001 |
| Israel | 36.34 | -0.70% | <0.001 |
| Japan | 36.88 | -1.24% | <0.001 |
| Jordan | 21.94 | -0.78% | <0.001 |
| Kazakhstan | 103.42 | -1.61% | <0.001 |
| Kuwait | 30.05 | -0.98% | <0.001 |
| Kyrgyzstan | 721.34 | 0.01% | <0.001 |
| Lao People's Democratic Republic | 602.35 | -0.02% | 0.319 |
| Lebanon | 31.83 | -1.15% | <0.001 |
| Malaysia | 545.99 | 0.07% | <0.001 |
| Maldives | 502.78 | -0.07% | <0.001 |
| Mongolia | 748.64 | -0.01% | 0.054 |
| Myanmar | 584.25 | -0.15% | <0.001 |
| Nepal | 534.10 | 0.13% | <0.001 |
| Oman | 32.01 | -0.18% | <0.001 |
| Pakistan | 770.70 | 0.25% | <0.001 |
| Palestine | 525.10 | 0.06% | <0.001 |
| Philippines | 398.21 | 0.30% | <0.001 |
| Qatar | 37.79 | -1.05% | <0.001 |
| Republic of Korea | 25.08 | -0.56% | <0.001 |
| Saudi Arabia | 35.91 | -0.65% | <0.001 |
| Singapore | 28.30 | -2.21% | <0.001 |
| Sri Lanka | 77.40 | -0.67% | <0.001 |
| Syrian Arab Republic | 521.39 | 0.13% | <0.001 |
| Tajikistan | 741.95 | 0.04% | <0.001 |
| Thailand | 481.42 | -0.06% | <0.001 |
| Timor-Leste | 522.78 | 0.13% | <0.001 |
| Turkey | 27.99 | -0.54% | <0.001 |
| Turkmenistan | 718.11 | 0.01% | 0.005 |
| United Arab Emirates | 511.64 | -0.01% | 0.103 |
| Uzbekistan | 746.93 | 0.24% | <0.001 |
| Viet Nam | 119.78 | 0.47% | <0.001 |
| Yemen | 639.56 | 0.26% | <0.001 |

Appendix table 4: Rheumatic heart disease DALYs per 100 000 population in 2019 and its average annual percentage change in the period 1990-2019

| Location Name | Age-standardized DALYs (per 100 000 population), 2019 | Average annual percent change (95% CI), 1990-2019 | P-value |
| --- | --- | --- | --- |
| Global | 132.88 | -2.60% | <0.001 |
| Asia | 175.20 | -2.80% | <0.001 |
| Central Asia | 161.24 | -2.20% | <0.001 |
| East Asia | 94.12 | -5.10% | <0.001 |
| High-income Asia Pacific | 13.20 | -3.60% | <0.001 |
| South Asia | 348.46 | -2.10% | <0.001 |
| Southeast Asia | 58.53 | -2.60% | <0.001 |
| Afghanistan | 210.71 | -2.20% | <0.001 |
| Armenia | 111.00 | -3% | <0.001 |
| Azerbaijan | 95.52 | -2.20% | <0.001 |
| Bahrain | 25.72 | -3% | <0.001 |
| Bangladesh | 105.36 | -1.80% | <0.001 |
| Bhutan | 338.50 | -2.70% | <0.001 |
| Brunei Darussalam | 58.27 | -2.30% | <0.001 |
| Cambodia | 119.69 | -3.40% | <0.001 |
| China | 93.73 | -5.10% | <0.001 |
| Cyprus | 41.41 | -3.80% | <0.001 |
| Democratic People's Republic of Korea | 206.87 | -1.50% | <0.001 |
| Georgia | 182.30 | 0.10% | 0.757 |
| India | 350.70 | -2.20% | <0.001 |
| Indonesia | 26.05 | -3.70% | <0.001 |
| Iran (Islamic Republic of) | 61.29 | -2.70% | <0.001 |
| Iraq | 64.61 | -3.60% | <0.001 |
| Israel | 33.30 | -2.30% | <0.001 |
| Japan | 14.13 | -3.40% | <0.001 |
| Jordan | 12.68 | -4.30% | <0.001 |
| Kazakhstan | 68.09 | -4.80% | <0.001 |
| Kuwait | 12.01 | -5.10% | <0.001 |
| Kyrgyzstan | 148.61 | -3.40% | <0.001 |
| Lao People's Democratic Republic | 168.46 | -2.30% | <0.001 |
| Lebanon | 24.60 | -4% | <0.001 |
| Malaysia | 52.65 | -3.90% | <0.001 |
| Maldives | 56.37 | -4.70% | <0.001 |
| Mongolia | 175.53 | -2.70% | <0.001 |
| Myanmar | 103.49 | -2.90% | <0.001 |
| Nepal | 358.56 | -2.50% | <0.001 |
| Oman | 12.93 | -4.30% | <0.001 |
| Pakistan | 542.04 | -1.10% | <0.001 |
| Palestine | 41.60 | -2.30% | <0.001 |
| Philippines | 126.91 | 1.30% | <0.001 |
| Qatar | 18.11 | -4.90% | <0.001 |
| Republic of Korea | 8.20 | -4.30% | <0.001 |
| Saudi Arabia | 29.83 | -4.20% | <0.001 |
| Singapore | 10.10 | -6.50% | <0.001 |
| Sri Lanka | 26.12 | -4.40% | <0.001 |
| Syrian Arab Republic | 78.17 | -5.40% | <0.001 |
| Tajikistan | 157.11 | -2.90% | <0.001 |
| Thailand | 38.86 | -5% | <0.001 |
| Timor-Leste | 133.79 | -1.40% | <0.001 |
| Turkey | 15.20 | -3.90% | <0.001 |
| Turkmenistan | 129.67 | -2.50% | <0.001 |
| United Arab Emirates | 108.70 | -2.90% | <0.001 |
| Uzbekistan | 240.27 | -1.70% | <0.001 |
| Viet Nam | 47.27 | -3.50% | <0.001 |
| Yemen | 148.06 | -2.20% | <0.001 |

Appendix table 5: Rheumatic heart disease YLLs per 100 000 population in 2019 and its average annual percentage change in the period 1990-2019

| Location Name | Age-standardized DALYs (per 100 000 population), 2019 | Average annual percent change (95% CI), 1990-2019 | P-value |
| --- | --- | --- | --- |
| Global | 25.21 | 0.50% | <0.001 |
| Asia | 23.25 | 0.20% | <0.001 |
| Central Asia | 29.88 | 0.20% | <0.001 |
| East Asia | 20.24 | -0.40% | <0.001 |
| High-income Asia Pacific | 2.00 | -1.10% | <0.001 |
| South Asia | 31.49 | 0.10% | <0.001 |
| Southeast Asia | 13.82 | 0.10% | <0.001 |
| Afghanistan | 24.19 | 0.10% | <0.001 |
| Armenia | 38.46 | -0.10% | <0.001 |
| Azerbaijan | 35.05 | -0.10% | <0.001 |
| Bahrain | 2.07 | -0.70% | <0.001 |
| Bangladesh | 26.66 | 0.20% | <0.001 |
| Bhutan | 28.00 | 0% | 0.024 |
| Brunei Darussalam | 3.60 | -0.90% | <0.001 |
| Cambodia | 20.72 | 0% | 0.316 |
| China | 20.41 | -0.40% | <0.001 |
| Cyprus | 2.08 | -1.40% | <0.001 |
| Democratic People's Republic of Korea | 21.23 | 0% | 0.017 |
| Georgia | 37.03 | 0% | <0.001 |
| India | 31.28 | 0.10% | <0.001 |
| Indonesia | 7.26 | 0.30% | <0.001 |
| Iran (Islamic Republic of) | 24.62 | 0.10% | <0.001 |
| Iraq | 25.28 | 0% | <0.001 |
| Israel | 2.30 | -0.70% | <0.001 |
| Japan | 2.19 | -1.10% | <0.001 |
| Jordan | 1.28 | -0.60% | <0.001 |
| Kazakhstan | 5.01 | -1.60% | <0.001 |
| Kuwait | 1.66 | -0.80% | <0.001 |
| Kyrgyzstan | 34.71 | 0% | 0.093 |
| Lao People's Democratic Republic | 28.92 | 0% | 0.838 |
| Lebanon | 1.86 | -0.90% | <0.001 |
| Malaysia | 26.28 | 0.10% | <0.001 |
| Maldives | 24.34 | -0.10% | <0.001 |
| Mongolia | 36.10 | 0% | 0.68 |
| Myanmar | 27.97 | -0.20% | <0.001 |
| Nepal | 25.69 | 0.10% | <0.001 |
| Oman | 1.62 | -0.10% | 0.014 |
| Pakistan | 37.70 | 0.20% | <0.001 |
| Palestine | 24.98 | 0.10% | <0.001 |
| Philippines | 19.39 | 0.30% | <0.001 |
| Qatar | 2.14 | -1% | <0.001 |
| Republic of Korea | 1.40 | -0.50% | <0.001 |
| Saudi Arabia | 2.08 | -0.50% | <0.001 |
| Singapore | 1.54 | -2% | <0.001 |
| Sri Lanka | 3.73 | -0.70% | <0.001 |
| Syrian Arab Republic | 25.42 | 0.10% | <0.001 |
| Tajikistan | 35.65 | 0% | <0.001 |
| Thailand | 23.39 | 0% | 0.005 |
| Timor-Leste | 25.14 | 0.10% | <0.001 |
| Turkey | 1.58 | -0.60% | <0.001 |
| Turkmenistan | 34.23 | 0% | 0.27 |
| United Arab Emirates | 24.80 | 0% | 0.015 |
| Uzbekistan | 35.67 | 0.20% | <0.001 |
| Viet Nam | 5.86 | 0.40% | <0.001 |
| Yemen | 30.62 | 0.30% | <0.001 |

Appendix table 6: Selected covariates for CODEm models

| Covariate | Transformation | Level | Direction |
| --- | --- | --- | --- |
| Rheumatic heat disease SEV scalar | None | 1 | 1 |
| Improved water (proportion) | None | 1 | -1 |
| Malnutrition | None | 1 | 1 |
| Sanitation (proportion with access) | None | 1 | -1 |
| Healthcare access and quality index | None | 2 | -1 |
| LDI | Log | 3 | -1 |
| SDI | None | 3 | -1 |
| Education (years per capita) | None | 3 | -1 |


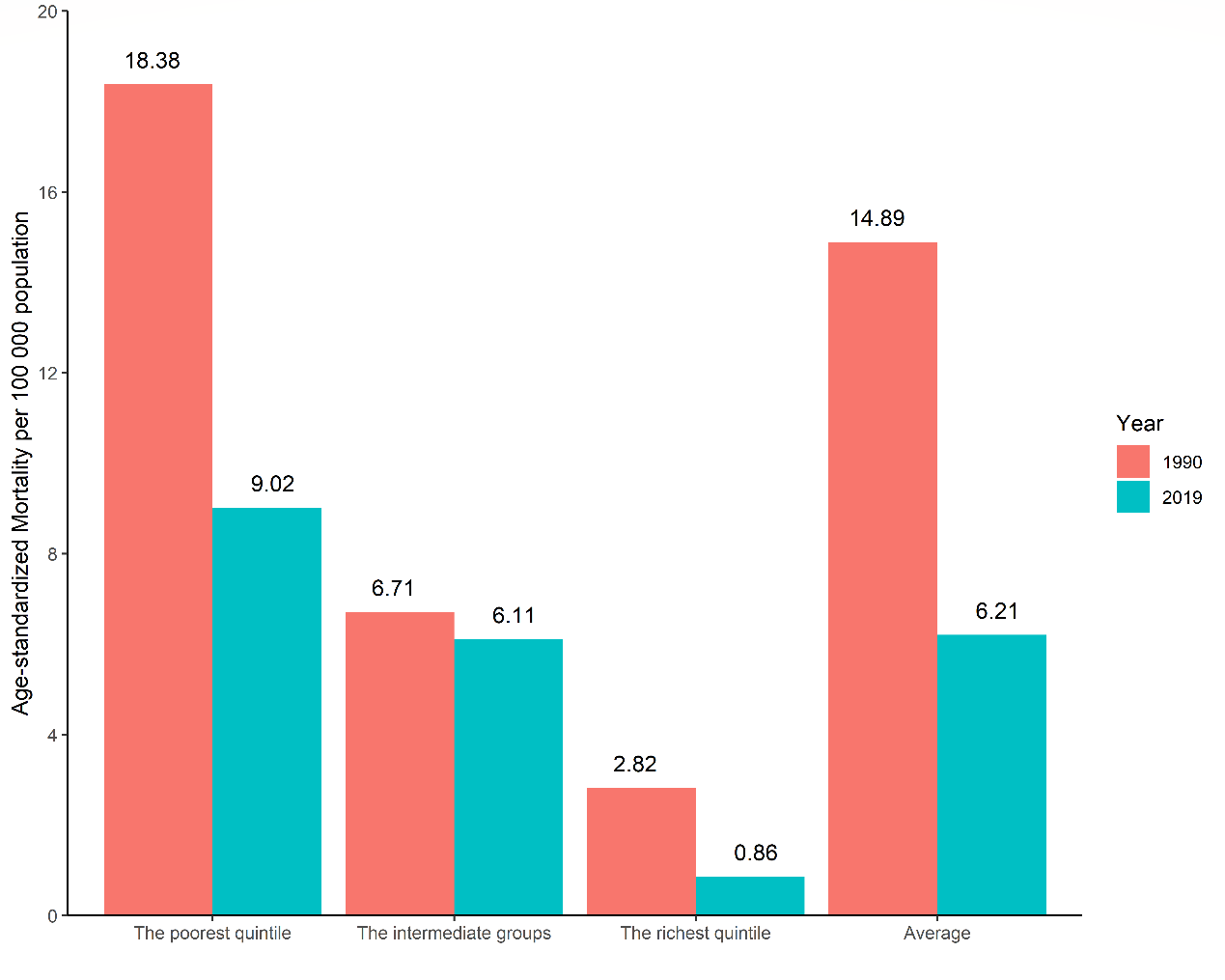


Appendix figure 1: Age-standardized mortality per 100 000 population in different income groups


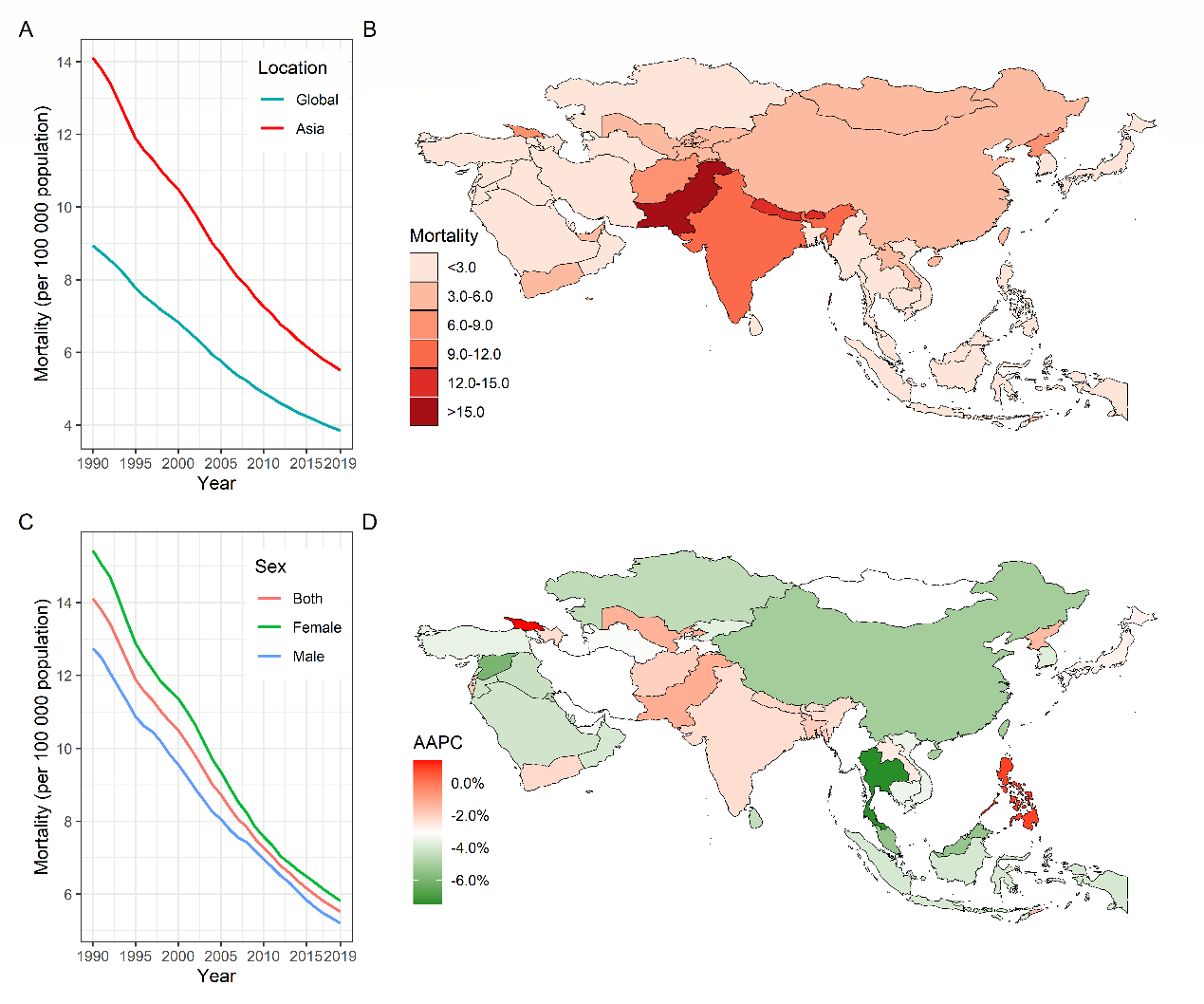


Appendix figure 2: Age-standardized mortality per 100 000 in 2019 and its AAPC of RHD for Asian 48 countries and territories. Age-standardized mortality of RHD per 100 000 population from 1990 through 2019 stratified by region (A) or sex(C). Age-standardized mortality of RHD per 100 000 population by country and territory, in 2019 (B). The relative changes in age-standardized mortality of RHD by country and territory from 1990 through 2019 (D). RHD: rheumatic heart disease.


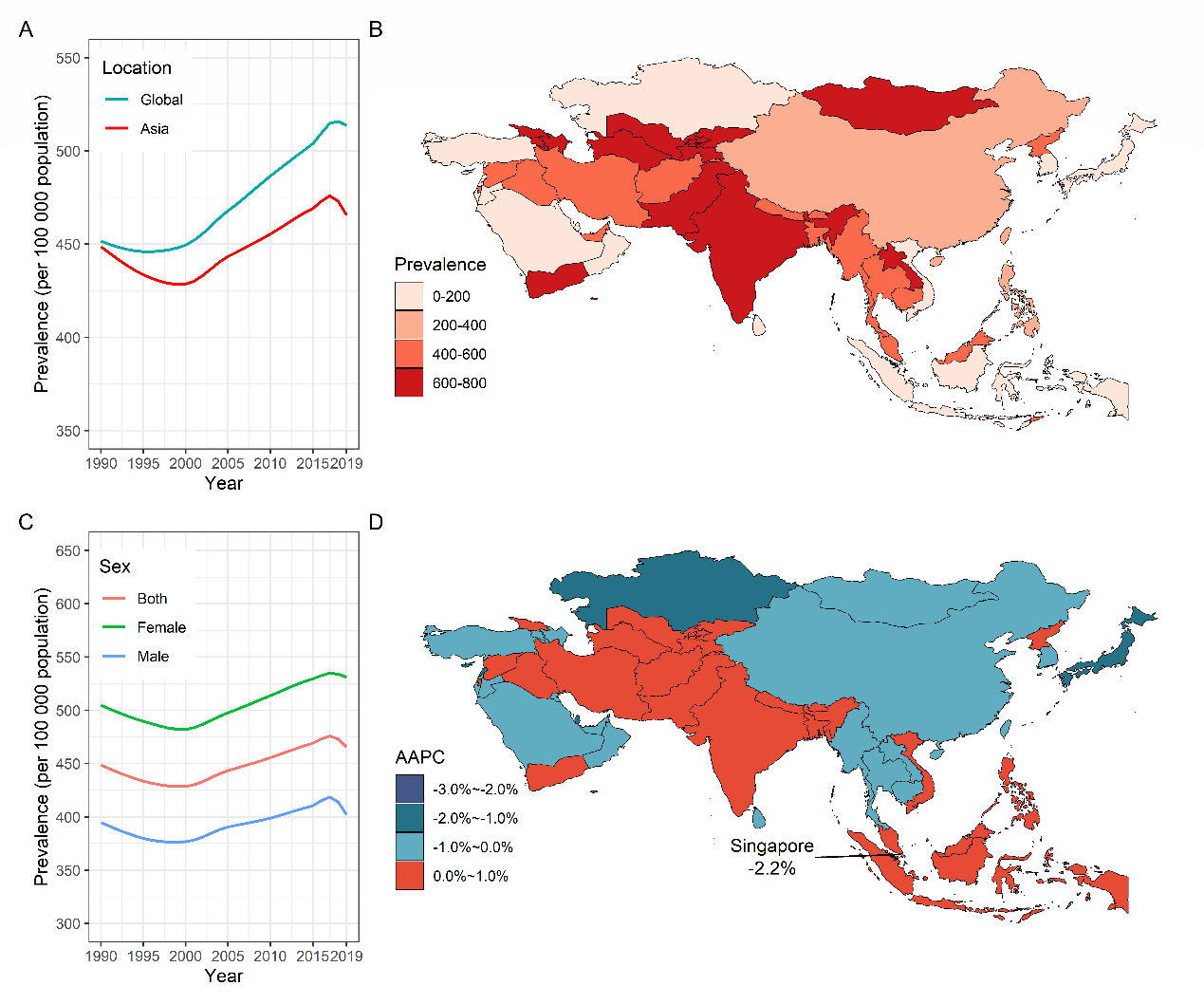


Appendix figure 3: Age-standardized prevalence rate per 100 000 in 2019 and its AAPC of RHD for Asian 48 countries and territories. Age-standardized prevalence of RHD per 100 000 population from 1990 through 2019 stratified by region (A) or sex(C). Age-standardized prevalence of RHD per 100 000 population by country and territory, in 2019 (B). The relative changes in age-standardized prevalence of RHD by country and territory from 1990 through 2019 (D). RHD: rheumatic heart disease.


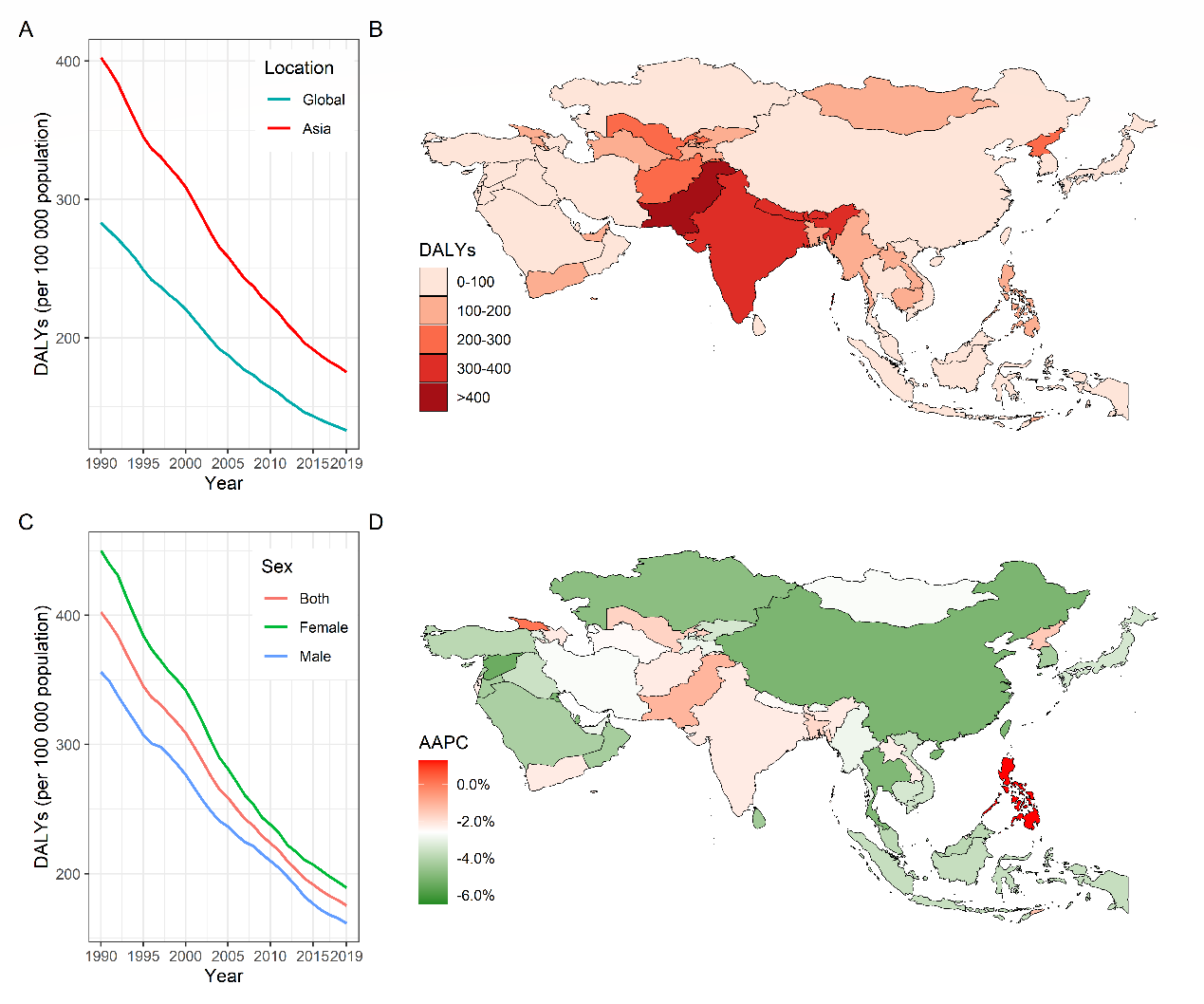


Appendix figure 4: Age-standardized DALYs per 100 000 in 2019 and its AAPC of RHD for Asian 48 countries and territories. Age-standardized DALYs of RHD per 100 000 population from 1990 through 2019 stratified by region (A) or sex(C). Age-standardized DALYs of RHD per 100 000 population by country and territory, in 2019 (B). The relative changes in age-standardized DALYs of RHD by country and territory from 1990 through 2019 (D). RHD: rheumatic heart disease.


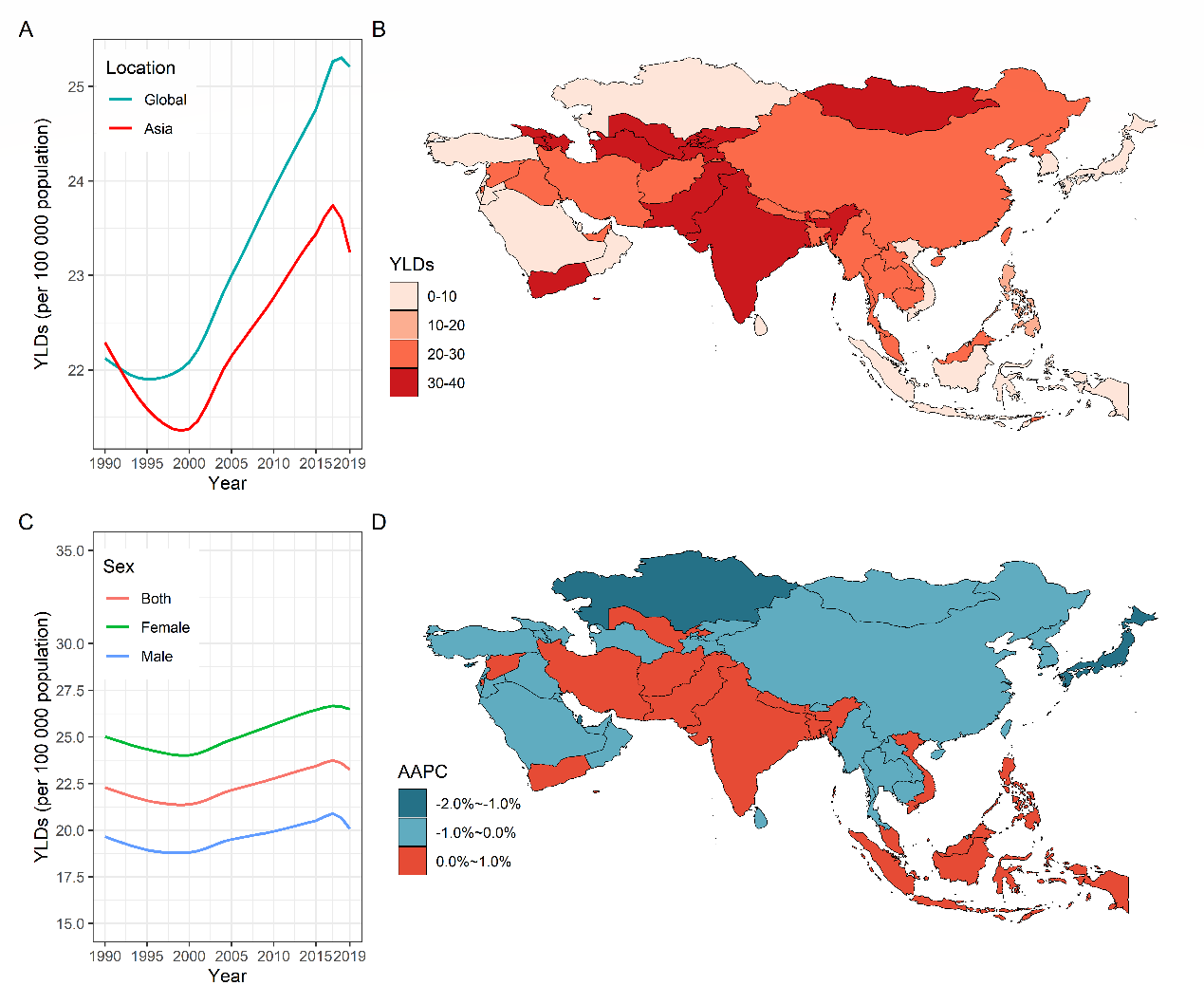


Appendix figure 5: Age-standardized YLDs per 100 000 in 2019 and its AAPC of RHD for Asian 48 countries and territories. Age-standardized YLDs of RHD per 100 000 population from 1990 through 2019 stratified by region (A) or sex(C). Age-standardized YLDs of RHD per 100 000 population by country and territory, in 2019 (B). The relative changes in age-standardized YLDs of RHD by country and territory from 1990 through 2019 (D). RHD: rheumatic heart disease.
